# The SARS-CoV-2 protein NSP2 enhances microRNA-mediated translational repression

**DOI:** 10.1242/jcs.261286

**Published:** 2023-10-11

**Authors:** Parisa Naeli, Xu Zhang, Patric Harris Snell, Susanta Chatterjee, Muhammad Kamran, Reese Jalal Ladak, Nick Orr, Thomas Duchaine, Nahum Sonenberg, Seyed Mehdi Jafarnejad

**Affiliations:** ^1^ Patrick G. Johnston Centre for Cancer Research, Queen's University Belfast, Belfast, BT9 7AE, UK; ^2^ Department of Biochemistry and Goodman Cancer Research Centre, McGill University, Montreal, H3A 1A3, Canada

**Keywords:** SARS-CoV-2, NSP2, microRNA, 4EHP, GIGYF2, mRNA translation

## Abstract

Viruses use microRNAs (miRNAs) to impair the host antiviral response and facilitate viral infection by expressing their own miRNAs or co-opting cellular miRNAs. miRNAs inhibit translation initiation of their target mRNAs by recruiting the GIGYF2–4EHP (or EIF4E2) translation repressor complex to the mRNA 5′-cap structure. We recently reported that the severe acute respiratory syndrome coronavirus 2 (SARS-CoV-2)-encoded non-structural protein 2 (NSP2) interacts with GIGYF2. This interaction is critical for blocking translation of the *Ifnb1* mRNA that encodes the cytokine interferon β, and thereby impairs the host antiviral response. However, it is not known whether NSP2 also affects miRNA-mediated silencing. Here, we demonstrate the pervasive augmentation of miRNA-mediated translational repression of cellular mRNAs by NSP2. We show that NSP2 interacts with argonaute 2 (AGO2), the core component of the miRNA-induced silencing complex (miRISC), via GIGYF2 and enhances the translational repression mediated by natural miRNA-binding sites in the 3′ untranslated region of cellular mRNAs. Our data reveal an additional layer of the complex mechanism by which SARS-CoV-2 and likely other coronaviruses manipulate the host gene expression program by co-opting the host miRNA-mediated silencing machinery.

## Teaser

**Summary:** SARS-CoV-2 non-structural protein 2 (NSP2) enhances the translational repression of host microRNA target mRNAs through interaction with GIGYF2 that forms a complex with the miRNA-induced silencing complex.

## INTRODUCTION

MicroRNAs (miRNAs) are small, ∼22-nucleotide-long non-coding RNAs that modulate the stability and translation efficiency of their target mRNAs. This is mediated by the miRNA-induced silencing complex (miRISC), an assembly of a miRNA, an argonaute (AGO) protein and other proteins, in which miRNA guides the complex to the target mRNA by sequence complementarity (Duchaine and Fabian, 2019). This leads to translational repression, followed by deadenylation and decapping of the mRNA, resulting in the exposure of the mRNA to exonuclease-mediated degradation (Djuranovic et al., 2012; Duchaine and Fabian, 2019; Fabian and Sonenberg, 2012; Naeli et al., 2022). The CCR4–NOT complex plays a key role in coordinating the intricate mechanism of regulation of mRNA translation and decay induced by miRNAs. Although miRNA-mediated deadenylation is achieved by the activity of the components of the catalytic subunits of the CCR4–NOT complex (CNOT6 or CNOT6L, and CNOT7 or CNOT8) (Lau et al., 2009; Piao et al., 2010), translational repression and decapping are engendered through the recruitment of several CCR4–NOT complex-binding proteins. We previously showed that the recruitment of the mRNA cap-binding eIF4E-homologue protein (EIF4E2 or 4EHP) by the CCR4–NOT complex is critical for the miRNA-mediated translational repression of target mRNAs (Chapat et al., 2017). 4EHP also forms a translational repressor complex with Grb10-interacting GYF protein 2 (GIGYF2) (Morita et al., 2012), which represses mRNA translation both in CCR4–NOT-dependent and -independent manners (Amaya Ramirez et al., 2018). The GIGYF2–4EHP complex is recruited by a variety of factors, including miRISC (Amaya Ramirez et al., 2018; Mayya et al., 2021), the RNA-binding protein tristetraprolin (TTP or ZFP36) (Fu et al., 2016) and the stalled ribosome-induced ribosome-associated quality control (RQC) mechanism via ZNF598 or EDF1 (Hickey et al., 2020), to repress translation.

Viruses use a variety of mechanisms to modulate host gene expression. A common strategy adopted by viruses involves the general shutdown of host mRNA translation, which allows redirecting host ribosomes toward viral mRNAs to express viral proteins (Hoang et al., 2021). These mechanisms include blocking cap-dependent translation initiation via sequestering or cleavage of the eukaryotic initiation factor 4G (eIF4G) (Belsham et al., 2000; Katsafanas and Moss, 2007), binding to and inducing the inhibition or degradation of poly(A)-binding proteins (PABPs) (Kuyumcu-Martinez et al., 2004), and binding to components of the eIF3 complex (Komarova et al., 2007). Many RNA viruses bypass the need for cap-dependent translation initiation by using an internal ribosome entry site (IRES) in the 5′ untranslated regions (UTRs) of the viral mRNA to enable translation in a ‘cap-independent’ manner (Hoang et al., 2021). In addition to shutdown of general cap-dependent translation, viruses also employ ‘targeted’ impairment of the homeostasis and proinflammatory responses of the host cell by changing the expression of miRNAs that target specific host mRNAs (Bhanja Chowdhury et al., 2012; Zhang et al., 2021). Furthermore, certain viruses express their own miRNAs that target cellular mRNAs (Pawlica et al., 2021). Conversely, host cells also express miRNAs that can interfere with viral infection by targeting viral mRNA or silencing anti-inflammatory factors (Potenza et al., 2011). Therefore, the precise regulation of miRNA-mediated silencing mechanisms is important for the viral infection as well as the host antiviral immune response.

We recently reported that the severe acute respiratory syndrome coronavirus 2 (SARS-CoV-2)-encoded non-structural protein 2 (NSP2) functions as a repressor of cellular mRNA translation via direct binding and stabilization of the GIGYF2–4EHP complex (Xu et al., 2022). However, it is not known whether and how NSP2 affects miRNA-mediated silencing, which also uses the GIGYF2–4EHP complex for translational repression of target mRNAs. Here, we provide evidence of a pervasive effect of NSP2 on miRNA-mediated silencing. We show that, through binding to GIGYF2, NSP2 also interacts with components of miRISC and enhances the translational repression of their target mRNAs by miRNAs.

## RESULTS

### NSP2 interacts with components of miRISC and enhances miRNA-mediated translational repression of target mRNA

**Fig. 1.**
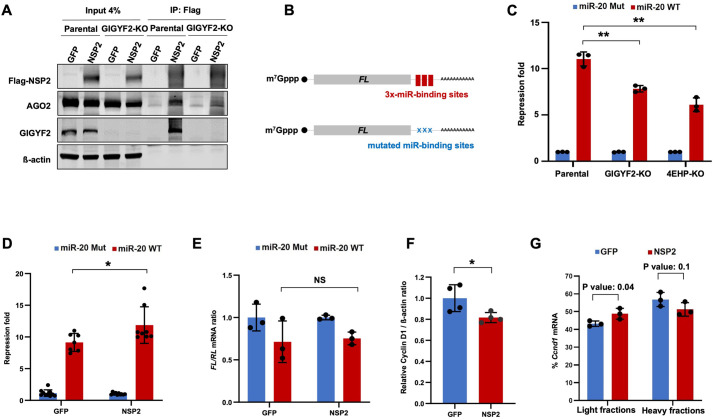
**NSP2 interacts with miRISC and enhances *miR-20a*-induced translational silencing.** (A) Parental and GIGYF2-KO HEK293 cells were co-transfected with Flag–GFP or Flag–NSP2 plasmids. 24 h later, proteins were immunoprecipitated (IP) from cell lysates using the anti-Flag antibody and western blotting was performed with the specified antibodies. Images are representative of three independent experiments. (B) Schematic representation of the firefly luciferase (*FL*) reporter with 3× *miR-20a*-binding sites (WT) in its 3′ UTR and the control *FL* reporter wherein all three *miR-20a*-binding sites were mutated (Mut). (C) Parental, GIGYF2-KO, and 4EHP-KO HEK293 cells were co-transfected with WT or Mut *FL*-miR-20 reporter, along with the Renilla luciferase (*RL*) reporter as control. (D) HEK293 cells were transfected with *FL*-miR-20 WT reporter or *FL*-miR-20 Mut reporter (control) and either Flag–GFP or Flag–NSP2 plasmids. For C,D, *FL* and *RL* activities were measured 24 h after transfection. *FL* values were normalized against *RL* levels, and repression fold was calculated for the *FL*-miR-20 WT relative to *FL*-miR-20 Mut level for each population. (E) RT-qPCR analysis of *FL*-miR-20 WT relative to *FL*-miR-20 Mut expression in Flag–GFP- or Flag–NSP2-expressing HEK293 cells, 24 h after transfection. (F) Quantitation of western blot analysis of cyclin D1 expression in lysates derived from HEK293 cells expressing Flag–NSP2 or Flag–GFP2 as control (see Fig. S1D for western blot images). Samples were collected from four independent biological replicates. β-actin was used as a loading control. The measured intensity of cyclin D1 bands were normalized to the corresponding β-actin bands. (G) RT-qPCR analysis of *Ccnd1* in sucrose fractions derived from polysome profiling in HEK293 cells expressing Flag–NSP2 or Flag–GFP as control (see Fig. S1F for the polysome profiling graph). Data are presented as mean±s.d. (*n*≥3). NS, not significant; *P<0.05; **P<0.01 (two-tailed unpaired Student's *t*-test).

To investigate a possible function for the interaction between NSP2 and the GIGYF2–4EHP complex on miRNA-mediated silencing, we first used co-immunoprecipitation, which showed that in addition to GIGYF2, NSP2 also co-precipitates with AGO2, the core component of miRISC in HEK293 cells (Fig. S1A). Importantly, CRISPR–Cas9-mediated knockout of GIGYF2 abrogated the pulldown of AGO2 by NSP2 (Fig. 1A). This indicates that the interaction between NSP2 and AGO2 is indirect and mediated by GIGYF2, which was previously shown to directly bind to NSP2 (Gupta et al., 2021, preprint) as well as the TNRC6 subunits of miRISC (Sobti et al., 2023).

We next examined the impact of NSP2 on the repression activity of miRNAs on a reporter mRNA that contains miRNA-binding sites. A recent analysis of dysregulated host miRNAs revealed *miR-20a* as one of the most significantly upregulated miRNAs in SARS-CoV-2-infected cells (Liu et al., 2022), likely due to its immunosuppressive effects (Cox et al., 2010; Reddycherla et al., 2015). We used a dual luciferase reporter system in which the firefly luciferase (*FL*) mRNA contains 3× *miR-20a*-binding sites within its 3′ UTR (miR-20 WT; Fig. 1B). As a control, a similar reporter was used in which all three miRNA-binding sites were mutated (miR-20 Mut; Fig. 1B). Expression of Renilla luciferase (*RL*) was used for normalization. We first determined whether the repression of the *FL* reporter by *miR-20a* is mediated by GIGYF2 and 4EHP by transfecting the parental, GIGYF2-knockout (KO) and 4EHP-KO HEK293 cells with miR-20 WT or miR-20 Mut reporters (Fig. S1B). Measurement of luciferase activity 24 h after transfection revealed a substantial and significant de-repression of the miR-20 WT reporter in GIGYF2-KO and 4EHP-KO cells compared with that in the parental cells (8-, 6- and 11-fold repression respectively; *P*<0.01; Fig. 1C). This result demonstrates that *miR-20a* represses the expression of the target mRNA in a GIGYF2- and 4EHP-dependent manner.

To examine the effect of NSP2 on *miR-20a*-induced repression, HEK293 cells were co-transfected with the miR-20 WT or miR-20 Mut reporters and plasmids expressing Flag–NSP2 or Flag–GFP as control. We observed that although miR-20 WT was significantly repressed compared with miR-20 Mut in GFP-expressing cells, co-expression with NSP2 further augmented *miR-20a*-mediated repression by ∼30% (9- and 11.9-fold for GFP- and NSP2-expressing cells, respectively; *P*<0.05; Fig. 1D; Fig. S1C). Following translational repression, miRNAs induce the degradation of their target mRNAs (Naeli et al., 2022). To test whether NSP2-enhanced *miR-20a*-induced silencing is due to augmented mRNA degradation, we measured the mRNA abundance of the *FL* and *RL* reporters by real-time quantitative PCR (RT-qPCR). This experiment revealed no significant difference in reporter mRNA expression between GFP- and NSP2-expressing cells (*P*>0.05; Fig. 1E). Consistently, we also observed an average of 19% reduced expression of cyclin D1 (CCND1), a natural target of *miR-20a* (Yu et al., 2008), at the protein level upon overexpression of NSP2 (*P*<0.05; Fig. 1F; Fig. S1D), in the absence of a detectable effect on *Ccnd1* mRNA expression (Fig. S1E). We further tested the changes in general mRNA translation upon NSP2 expression in HEK293 cells by polysome profiling, which assesses the association of ribosomes with mRNAs and provides valuable information about the translational status of mRNAs. We observed that expression of Flag–NSP2 did not have a tangible impact on the general association of mRNAs with polysomes, compared with that in Flag–GFP-expressing cells (Fig. S1F,G). However, NSP2 expression induced a significant, albeit small, enrichment for *Ccnd1* mRNA in the light sucrose fractions, which typically contain translationally repressed mRNAs (Fig. 1G). These data suggest that NSP2 affects mRNA translation in a transcript-specific manner and are consistent with our previous observations that GIGYF2 and 4EHP, the cellular interacting partners of NSP2, also affect mRNA translation in a transcript-specific manner and that their depletion does not have a tangible impact on general mRNA translation (Jafarnejad et al., 2018; Xu et al., 2022).

### NSP2-induced translational repression by miRNAs is pervasive

**Fig. 2.**
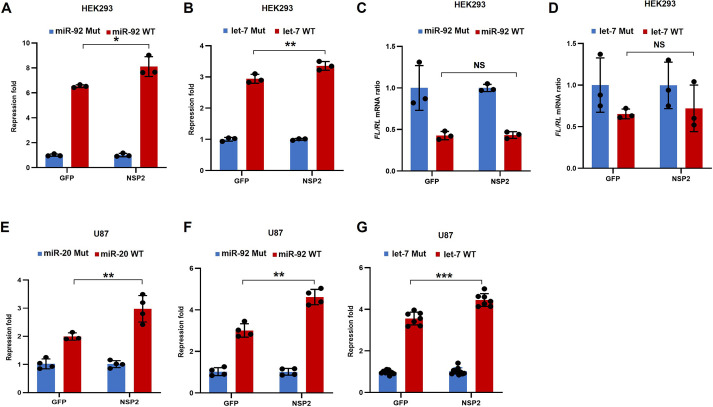
**Pervasive effects of NSP2 on miRNA-mediated silencing.** (A,B) HEK293 cells were co-transfected with WT or Mut *FL*-miR-92 reporter (A) and WT or Mut *FL*-let-7 reporter (B), along with *RL* as control. Luciferase activity was measured 24 h after transfection. *FL* values were normalized against *RL* levels, and repression fold was calculated for the WT relative to the respective Mut levels for each condition. (C) RT-qPCR analysis of *FL*-miR-92 WT relative to *FL*-miR-92 Mut expression in Flag–GFP- or Flag–NSP2-expressing HEK293 cells, 24 h after transfection. (D) RT-qPCR analysis of *FL*-let-7 WT relative to *FL*-let-7 Mut expression in Flag–GFP- or Flag–NSP2-expressing HEK293 cells, 24 h after transfection. (E–G) U87 cells were co-transfected with WT or Mut *FL*-miR-20 (E), *FL*-miR-92 (F) or *FL*-let-7 reporter (G) along with Flag–NSP2 or Flag–GFP as a control. 24 h later, cells were lysed and luciferase activity was measured. *FL* values were normalized against *RL* levels, and repression fold was calculated for the WT relative to the respective Mut version of the reporter for each condition. Data are presented as mean±s.d. (*n*≥3). NS, not significant; *P<0.05; **P<0.01; ***P<0.001 (two-tailed unpaired Student's *t*-test).

Hitherto, our data have revealed that NSP2 augments *miR-20a*-mediated repression. To investigate whether NSP2 also augments the translational repression by other miRNAs, we used two other abundant miRNAs: *let-7a*, which is also upregulated in SARS-CoV-2-infected cells (Nersisyan et al., 2020), and *miR-92*, the expression of which is not known to be modulated by SARS-CoV-2 (Liu et al., 2022). Similar to the miR-20 reporter (Fig. 1B), we used the dual luciferase reporter system in which the *FL* mRNA contained either 3× *miR-92*-binding sites (miR-92 WT) or 3× *let-7a*-binding sites (let-7 WT) within its 3′ UTR. As controls, similar reporters were used in which all three miRNA-binding sites were mutated (miR-92 Mut and let-7 Mut, respectively). NSP2 overexpression resulted in enhanced repression by *miR-92* (6- and 8-fold for GFP and NSP2, respectively; *P*<0.05; Fig. 2A; Fig. S2A) and *let-7a* (2.8- and 3.3-fold for GFP and NSP2, respectively; *P*<0.01; Fig. 2B; Fig. S2B) in HEK293 cells. As with *miR-20a*, no significant changes in the mRNA levels of miR-92 WT and let-7 WT reporters were detected, demonstrating that NSP2-induced enhanced repression occurs at the translational level (Fig. 2C,D).

We next examined whether the observed NSP2-mediated enhanced translational repression by miRNAs in HEK293 cells could also be observed in another cell type, the U87 human glioblastoma cell line. Consistent with the results in HEK293 cells, co-expression of NSP2 resulted in significantly enhanced repression by all three tested miRNAs (∼50%, ∼50% and ∼30% increase for the miR-20, miR-92, and let-7 reporters, respectively; Fig. 2E–G; Fig. S2C–E). Taken together, these data indicate a pervasive role for NSP2 in augmenting miRNA-mediated silencing.

### NSP2 regulates miRNA-mediated silencing of natural 3′ UTR sequences

**Fig. 3.**
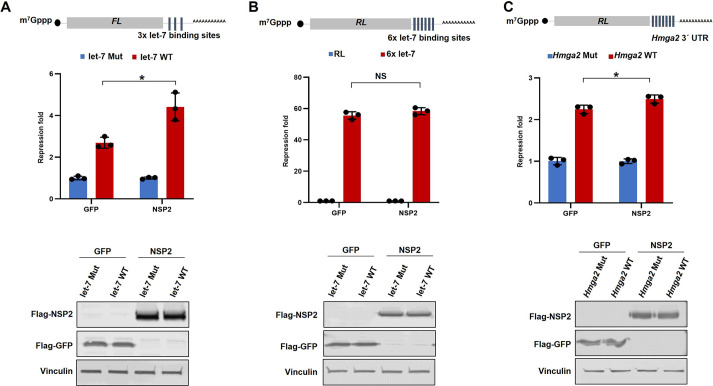
**NSP2 enhances the *let-7a*-induced silencing of a reporter with a natural 3′ UTR.** (A) Top: HEK293T cells were co-transfected with the *FL*-3×-let-7 (WT) reporter or the control version wherein the three *let-7a*-binding sites were mutated (Mut), along with Flag–NSP2 or Flag–GFP plasmid as a control. Luciferase activity was measured 24 h after transfection. *FL* values were normalized against *RL* levels, and repression fold was calculated for the WT relative to Mut reporter for each population. Bottom: western blots showing the expression of NSP2, GFP and vinculin (loading control), corresponding to the top panel. (B) Top: HEK293T cells were co-transfected with the *RL*-6×-let-7 reporter or the control *RL* with no *let-7a*-binding sites, along with *FL* control and Flag–NSP2 or Flag–GFP plasmid as a control. Luciferase activity was measured 24 h after transfection. *RL* values were normalized against *FL* levels, and repression fold was calculated for the *RL*-6×-let-7 relative to the *RL* control reporter for each population. Bottom: western blots showing the expression of NSP2, GFP and vinculin (loading control) corresponding to the top panel. (C) Top: HEK293 cells were co-transfected with *RL*-*Hmga2* 3′ UTR WT or *RL*-*Hmga2* 3′ UTR Mut reporter, wherein all seven *let-7a*-binding sites were mutated, *FL* plasmid (control), and either Flag–GFP or Flag–NSP2 plasmids. Luciferase activity was measured at 24 h post transfection. *RL* values were normalized against *FL* levels, and repression fold was calculated for *RL*-*Hmga2* 3′ UTR WT relative to the *RL*-*Hmga2* 3′ UTR Mut reporter for each condition. Bottom: western blots showing the expression of NSP2, GFP and vinculin (loading control) corresponding to the top panel. Data are presented as mean±s.d. (*n*≥3). NS, not significant; *P<0.05 (two-tailed unpaired Student's *t*-test).

Although it bolsters our findings that NSP2 interacts with the GIGYF2–4EHP complex, a recent study concluded that this interaction impairs the silencing of target mRNAs induced by *let-7a* miRNA (Zou et al., 2022). We noted that, whereas in our study, we used a luciferase reporter with 3× *let-7a*-binding sites (Fig. 2B,G), Zou et al. (2022) used a reporter with 6× *let-7a*-binding sites. We therefore performed an experiment in which we compared the effects of NSP2 overexpression on reporters with 3× or 6× *let-7a*-binding sites in HEK293T cells (Fig. 3A,B). As expected, the repression of the reporter with 3× *let-7a*-binding sites increased by ∼60% (2.7- and 4.4-fold in GFP- and NSP2-expressing cells, respectively; *P*<0.05; Fig. 3A). However, NSP2 overexpression had no significant effect on the reporter with 6× *let-7a*-binding sites (*P*>0.05; Fig. 3B). We reason that this difference might be due to a saturation of the repression mediated by the highly potent 6× *let-7a*-binding sites (>50-fold repression), which could not be further enhanced by NSP2. In contrast, the 3× let-7 reporter was repressed 10-fold less than the 6× let-7 reporter (<5-fold), which provides a more accurate and physiologically relevant assay for measurement of the impacts of NSP2 on miRNA-mediated silencing.

To further corroborate this conclusion, we tested a reporter fused to the natural (WT) 3′ UTR of *Hmga2* mRNA, an endogenous target of *let-7a* miRNA (Mayr et al., 2007), or a modified version bearing point mutations disrupting all seven *let-7a*-binding sites (Fig. 3C), in the presence of NSP2 or a GFP control in HEK293 cells. Although the reporter containing the WT *Hmga2* mRNA 3′ UTR was repressed by 2.2-fold compared with the mutated control reporter in GFP-expressing cells, this repression was significantly enhanced to 2.5-fold in NSP2-expressing cells (*P*<0.05; Fig. 3C). It should be noted that although this natural *Hmga2* mRNA 3′ UTR contains seven *let-7a*-binding sites, it induces considerably less repression compared with the 6× let-7 reporter (2-fold and 50-fold repression, respectively). This is likely due to the fact that the natural *Hmga2* 3′ UTR contains additional structures and regulatory elements such as RNA-binding protein (RBP)-binding sites (Busch et al., 2016; Degrauwe et al., 2016) that modulate miRNA-mediated silencing (Hausser et al., 2009) but are reflective of physiological repression.

### NSP2 augments AGO2-mediated translational repression

**Fig. 4.**
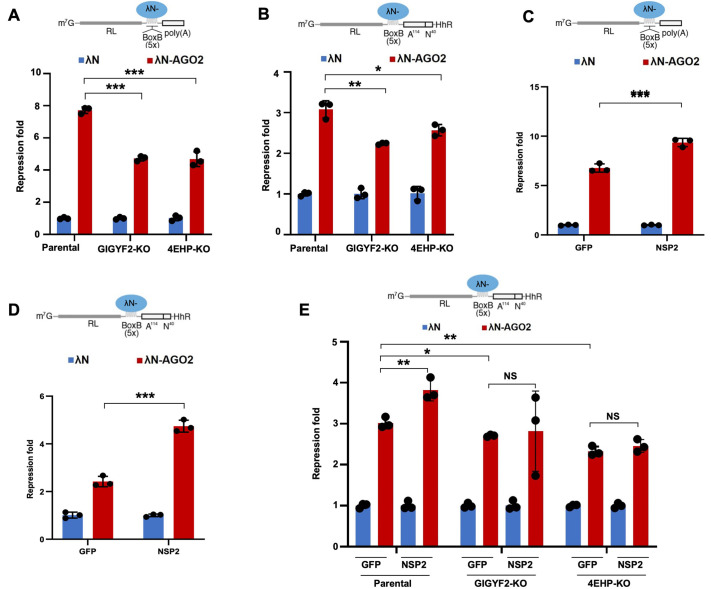
**NSP2 augments miRISC-mediated translational silencing.** (A) Top: schematic representation of the deadenylation-permissive *RL*-5BoxB reporter. Bottom: parental, GIGYF2-KO and 4EHP-KO HEK293 cells were co-transfected with *RL*-5BoxB reporter and either λN-AGO2 or λN-empty along with the *FL* plasmid (control). The cells were lysed after 24 h and luciferase activity was measured. *RL* values were normalized against *FL* levels, and repression fold was calculated for the λN-AGO2 relative to λN-empty level for each condition. (B) Top: schematic representation of the deadenylation-resistant *RL*-5BoxB-HhR reporter. Bottom: Parental, GIGYF2-KO, and 4EHP-KO HEK293 cells were co-transfected with *RL*-5BoxB-HhR reporter and either λN-AGO2 or λN-empty along with *FL* plasmid (control). The cells were lysed after 24 h and luciferase activity was measured. *RL* values were normalized against *FL* levels, and repression fold was calculated for the λN-AGO2 relative to λN-empty level for each condition. (C) HEK293 cells were co-transfected with the deadenylation-permissive *RL*-5BoxB reporter and either λN-AGO2 or λN-empty plasmid, along with *FL* plasmid (control) and either Flag–NSP2 or Flag–GFP plasmids. *RL* and *FL* luciferase activity was measured 24 h after transfection and *RL* values were normalized against *FL* levels. Repression fold was calculated for the λN-AGO2 relative to λN-empty level for each condition. (D) HEK293 cells were co-transfected with the deadenylation-resistant *RL*-5BoxB-HhR reporter and λN-AGO2 or λN-empty plasmid, along with *FL* plasmid (control) and either Flag–NSP2 or Flag–GFP plasmids. *RL* and *FL* luciferase activity was measured 24 h after transfection and *RL* values were normalized against *FL* levels. Repression fold was calculated for the λN-AGO2 relative to λN-empty level for each condition. (E) Parental, GIGYF2-KO and 4EHP-KO HEK293 cells were co-transfected with the deadenylation-resistant *RL*-5BoxB-HhR reporter and λN-AGO2 or λN-empty plasmid, along with *FL* plasmid (control) and either Flag–NSP2 or Flag–GFP plasmids. *RL* and *FL* luciferase activity was measured 24 h after transfection and *RL* values were normalized against *FL* levels. Repression fold was calculated for the λN-AGO2 relative to λN-empty level for each condition. Data are presented as mean±s.d. (*n*≥3). NS, not significant; *P<0.05; **P<0.01; ***P<0.001 (two-tailed unpaired Student's *t*-test).

To rule out the possible confounding contributions of changes in miRNA expression upon NSP2 overexpression on our reporter activities, we sought to assess the impact of NSP2 on miRISC activity in a design that is independent of miRNA species. For this, we used the LambdaN (λN):BoxB system to tether AGO2, which is the minimum required subunit for a functional miRISC (Rivas et al., 2005). A Renilla luciferase (*RL*) gene encoding five BoxB hairpins in its 3′ UTR (*RL*-5BoxB) was used to tether a λN–AGO2 fusion (Fig. 4A). λN–AGO2 repressed the *RL*-5BoxB reporter by 7.7-fold compared with the λN-empty control construct (Fig. 4A). Importantly, this repression was reduced to 4.7-fold in GIGYF2-KO and 4.6-fold in 4EHP-KO HEK293 cells (Fig. 4A; Fig. S3A), indicating the contribution of a GIGYF2–4EHP-dependent mechanism in AGO2-mediated repression. The prevailing model of miRNA-mediated silencing supports a successive progression from translational repression followed by degradation (decay) of the target mRNA (Naeli et al., 2022). To specifically dissect the impact on mRNA translation, we used a variation of a *RL*-5BoxB reporter that encodes a self-cleaving hammerhead ribozyme (HhR) at its 3′ end to generate an internalized poly(A) stretch of 114 nucleotides to prevent deadenylation and subsequent degradation of the reporter mRNA (Fig. 4B). Tethering of λN–AGO2 resulted in a significant repression of the *RL*-5BoxB-HhR reporter by 3.1-fold (Fig. 4B). Importantly, this repression was significantly reduced in GIGYF2-KO and 4EHP-KO cells (to 2.2-fold and 2.5-fold, respectively; Fig. 4B; Fig. S3B), indicating that the translational repression of the target mRNA by AGO2 is GIGYF2–4EHP dependent.

**Fig. 5.**
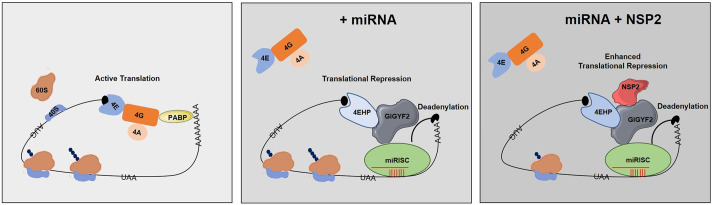
**NSP2 modulates cellular miRNA-mediated silencing through GIGYF2-mediated indirect interaction with miRISC.** Graphic illustration of the mechanism by which NSP2 modulates cellular miRNA-mediated silencing. The eIF4F complex consisting of eIF4E (4E), eIF4G (4G) and eIF4A (4A) facilitates the recruitment of the small ribosomal subunit and initiation of mRNA translation. PABP stabilizes the poly(A) tail and, through interaction with eIF4G, enables circularization of the mRNA that is believed to enhance translation efficiency (left). The GIGYF2–4EHP complex enables miRISC-mediated repression of cap-dependent mRNA translation by displacing the eIF4F complex from the 5′ cap (middle). Binding of NSP2 to GIGYF2 enhances the interaction between GIGYF2 and 4EHP and the miRNA-mediated translational repression without affecting mRNA deadenylation and/or stability (right).

Having established this model, we tested the impact of NSP2 on AGO2-mediated silencing. In contrast with the GFP control, overexpression of NSP2 significantly enhanced the λN–AGO2-induced repression of both the degradation-permissive *RL*-5BoxB (Fig. 4C; Fig. S3C) and the degradation-resistant *RL*-5BoxB-HhR reporters (Fig. 4D; Fig. S3D). Importantly, this NSP2-mediated enhanced repression of *RL*-5BoxB-HhR reporter mRNA by λN-AGO2 was abrogated in the GIGYF2-KO or 4EHP-KO cells (Fig. 4E; Fig. S3E). Taken together, these data provide strong evidence that NSP2 enhances translational repression by miRISC in a GIGYF2–4EHP-dependent manner (Fig. 5).

## DISCUSSION

Here, we describe a role for the SARS-CoV-2 NSP2 protein in regulation of miRNA-mediated translational repression. We demonstrate that via GIGYF2, NSP2 interacts with AGO2 and thereby augments miRNA-mediated translational repression of target mRNAs.

We previously showed that NSP2 facilitates SARS-CoV-2 viral replication by augmenting GIGYF2–4EHP-mediated repression of *Ifnb1* mRNA, which encodes the key cytokine IFN-β (Xu et al., 2022). Notably, *Ifnb1* mRNA contains the binding sites for multiple miRNAs including *let-7a*, *miR-34a*, *miR-26a* and *miR-145* (Witwer et al., 2010). Computational analyses identified a substantial number of potential miRNAs encoded by the SARS-CoV-2 genome, many of which were predicted to target mRNAs that encode proteins with important roles in immune regulatory processes such as NF-κB, JAK/STAT and TGFβ signalling pathways (Aydemir et al., 2021). Further studies empirically identified multiple miRNAs derived from the viral genome that impaired the host antiviral response by targeting the 3′ UTR of various mRNAs that encode IRF7, IRF9, STAT2 (Zhu et al., 2021) and interferon-stimulated genes (e.g. ISG15, MX1 and BATF2) (Morales et al., 2017; Pawlica et al., 2021; Singh et al., 2022). It is thus likely that enhanced translational repression of targets of both viral and host miRNA through the function of NSP2 serves to impair a host innate immune response against SARS-CoV-2 infection. However, in principle, NSP2 could also enhance the silencing mediated by antiviral miRNAs (Mahajan et al., 2009). To avoid the potential harmful effects of NSP2-induced repression by antiviral miRNAs, the virus can manipulate the expression pattern of antiviral miRNAs (Centa et al., 2021; Chow and Salmena, 2020; Gambardella et al., 2020; Liu et al., 2022; Plowman and Lagos, 2021). Analysis of the expression of 128 human miRNAs with potential to target the SARS-CoV-2 genome revealed their very low expression in lung epithelia (Chow and Salmena, 2020), which likely allows the virus to avoid the effects of antiviral miRNAs and replicate in these cells.

It is estimated that miRNAs target over 60% of human protein-coding mRNAs (Friedman et al., 2009) and affect important processes including development, cell proliferation, metabolism and maintenance of homeostasis (Vidigal and Ventura, 2015). Dysregulated miRNA expression and activity have been linked to diseases including cancer and metabolic disorders (Huang et al., 2019; Najafi-Shoushtari et al., 2010). Hence, control of miRNA-mediated translational repression by NSP2 during SARS-CoV-2 infection could have significant pathophysiological impacts. Importantly, NSP2, or NSP2-derived peptides that preserve the ability to augment the GIGYF2–4EHP complex, could potentially be used to modulate miRNA-mediated silencing, independent of SARS-CoV-2 infection. For instance, global miRNA expression is often downregulated in cancers (Hata and Lieberman, 2015), due to various reasons such as dysregulated expression of the miRNA biogenesis factor Dicer (Bandara et al., 2014; Jafarnejad et al., 2013; Merritt et al., 2008). It would be interesting to assess the potential effects of the enhancement of miRNA-mediated silencing by NSP2 on the tumorigenicity of cancer cells with reduced miRNA biogenesis capacity.

Although, on the transcriptome-wide scale, each miRNA can potentially target hundreds of mRNAs, miRNAs generally have relatively subtle impacts on the stability or translation of individual target mRNAs (Selbach et al., 2008). In addition, the miRNA-mediated silencing machinery has a limited capacity and is prone to saturation (Castanotto et al., 2007; Khan et al., 2009). This limited capacity for miRNA-mediated silencing machinery should be considered when interpreting data generated by transfection of ectopic miRNAs, reporter mRNAs with miRNA-binding sites, or when tethering components of miRISC. Congruent with this notion, NSP2 did not have a significant effect on a reporter mRNA bearing 6× *let-7a*-binding sites, which was repressed by ∼50-fold compared with the mutated control (Fig. 3B). In contrast, repression of the reporter with 3× *let-7a*-binding sites (Fig. 3A) or a reporter with the natural 3′ UTR of the *Hmga2* mRNA, which were both repressed by less than 3-fold, was significantly enhanced upon ectopic expression of NSP2 (Fig. 3C). This might also explain the discrepancy in the results that we obtained with multiple miRNA reporters (miR-20, miR-92 and let-7) as well as with the tethering of AGO2, compared with the study by Zou et al. (2022), who used the 6× let-7 reporter or tethering of the GW182 silencing domain that induced >40-fold repression.

In addition to mediating the miRNA-induced repression of the cap-dependent mRNA translation, the GIGYF2–4EHP complex can also take part in silencing of target mRNAs triggered by ribosome stalling (RQC) (Hickey et al., 2020) or RNA-binding proteins such as TTP (Fu et al., 2016). TTP and its paralogues ZFP36L1 and ZFP36L2 have significant roles in cancer (Turner et al., 2014) and regulation of the immune system (Turner et al., 2014) through binding and repressing the expression of mRNAs that contain A/U rich elements. A recent study also revealed the biological importance of RQC in neurological disorders (Lu, 2022). Future studies will assess the impact of NSP2 on the translational silencing mediated by RBPs that recruit the GIGYF2–4EHP complex, such as TTP and ZNF598, and the biological pathways they control.

## MATERIALS AND METHODS

### Cell lines and culture conditions

HEK293T (Thermo Fisher Scientific) and U87 cells (American Type Culture Collection) were cultured in Dulbecco's modified Eagle medium (DMEM; Gibco, 41965039) supplemented with 10% foetal bovine serum (FBS; Gibco, 10270106), 100 U/ml penicillin and 100 µg/ml streptomycin (Gibco, 5070063). 4EHP-KO and GIGYF2-KO HEK293 cells have been described previously (Xu et al., 2022; Zhang et al., 2021). Parental, 4EHP-KO and GIGYF2-KO HEK293 cells were maintained in DMEM supplemented with 10% FBS, 1% penicillin/streptomycin, 100 µg/ml zeocin (Invitrogen, 460509) and 15 µg/ml blasticidin (BioShop, BLA477). All cells were cultured at 37°C in a humidified atmosphere with 5% CO_2_ and regularly tested for the presence of mycoplasma contamination using a mycoplasma detection kit (Applied Biological Materials, G238).

### Plasmids and cloning

To generate the v5-tagged λN-AGO2 plasmid, the AGO2 coding sequence (CDS) was PCR-amplified using the pFRT/FLAG/HA-DEST EIF2C2 plasmid (Addgene #19888; Landthaler et al., 2008) as template and Q5 High-Fidelity DNA Polymerase (New England Biolabs, M0515). The AGO2 CDS was subsequently cloned into the pCI-neo-λN-v5 plasmid using the EcoRI and NotI sites. The pmiRGLO plasmid (Promega)-based miR-20, miR-92 and let-7 reporters and the corresponding mutant reporters have been described previously (Mayya and Duchaine, 2015). The *RL*-6× let-7 reporter has been described previously (Mathonnet et al., 2007). The pcDNA3-Flag-GFP and pcDNA3-Flag-NSP2 plasmids have been described previously (Xu et al., 2022). The *Hmga2* reporter has been described previously (Mayr et al., 2007).

### Dual luciferase reporter assay

For experiments with miRNAs reporters, 150×10^3^ HEK293 or 175×10^3^ U87 cells were seeded in 24-well plates and transfected the next day with 100 ng of Flag–NSP2 or Flag–GFP plasmids, and 10 ng of wild-type (WT) or mutant (Mut) versions of pmiRGLO-3×-let-7a, pmiRGLO-3×-miR-20a, or pmiRGLO-3×-miR-92 miRNA reporters using Lipofectamine 2000 (Invitrogen, 11668019). For experiments with the 6× let-7 reporter, 150×10^3^ HEK293T cells were seeded in a 24-well plate and were transfected the next day with 100 ng of Flag–NSP2 or Flag–GFP plasmids, and 20 ng of *RL*-6× let-7 and 5 ng of *FL* plasmids as control, using Lipofectamine 2000. The transfection medium was replaced with fresh DMEM containing 10% FBS 6 h after transfection. The cells were lysed 24 h after transfection and luciferase activity was measured with the Dual-Luciferase Reporter Assay kit (Promega, E1960) according to the manufacturer's instructions using a FLUOstar plate reader (BMG Labtech). Firefly luciferase (*FL*) activity was normalized to Renilla luciferase (*RL*) activity and the values are shown as repression fold relative to the control.

For AGO2 tethering, 150×10^3^ HEK293 cells were transfected in a 24-well plate with 5 ng of the *FL* plasmid, 20 ng of *RL*-5BoxB or *RL*-5boxB-A114-N40-HhR, 100 ng of v5-tagged λN-AGO2 or λN-empty and 100 ng of the plasmid encoding Flag–NSP2 or Flag–GFP using Lipofectamine 2000. Cells were lysed 24 h after transfection and luciferase activity was measured with the Dual-Luciferase Reporter Assay according to the manufacturer's instructions using the FLUOstar plate reader. *RL* activity was normalized to *FL* activity and the repression fold was calculated for λN-AGO2 relative to λN-empty control.

### RNA extraction and RT-qPCR

Total RNA was isolated using TRIzol reagent (Thermo Fisher Scientific, 15596026) according to the manufacturer's instructions. 1 µg purified total RNA was treated with DNase I (Thermo Fisher Scientific, EN0521), prior to reverse transcription using SuperScript III Reverse Transcriptase (Thermo Fisher Scientific, 18080085) and random hexamers. The following DNA oligonucleotides were used as primers for PCR reactions: pmiRGLO Fluc Forward, 5′-ACTTCGAGATGAGCGTTCGG-3′; pmiRGLO Fluc Reverse, 5′-CCAACACGGGCATGAAGAAC-3′; pci-neo Fluc Forward, 5′-GAGCACGGAAAGACGATGACGG-3′; pci-neo Fluc Reverse,: 5′-GGCCTTTATGAGGATCTCTCTG-3′; Rluc Forward, 5′-ATGGCTTCCAAGGTGTAC-3′; Rluc Reverse, 5′-TAGTTGATGAAGGAGTCCA-3′; CCND1 Forward, 5′-ACAAACAGATCATCCGCAAACAC-3′; CCND1 Reverse, 5′-TGTTGGGGCTCCTCAGGTTC-3′; β-actin Forward, 5′-ACAGAGCCTCGCCTTTGCC-3′; and β-actin Reverse, 5′-GATATCATCATCCATGGTGAGCTGG-3′. Quantitative PCR (qPCR) was performed on LightCycler 480 Instrument II (Roche) using the LightCycler 480 SYBR Green I Master mix (Roche, 04887352001) according to the manufacturer's protocol.

### Western blotting

For western blotting, cells were lysed in RIPA buffer (50 mM Tris-HCl pH 7.4, 150 mM NaCl, 2 mM EDTA, 1% NP-40 and 0.1% SDS) supplemented with protease inhibitors (Roche, 11836170001). SDS-PAGE gels were used for protein separation, followed by transfer onto PVDF membranes (Merck, IPFL00010). Blots were blocked with 5% bovine serum albumin at room temperature for 1 h and incubated with the following primary antibodies overnight at 4°C: mouse anti-v5 (Thermo Fisher Scientific, R96025; 1:2000), mouse anti-Flag M2 (Sigma-Aldrich, F3165; 1:2000), mouse anti-β-actin (Sigma-Aldrich, A5441; 1:1000), rabbit anti-GIGYF2 (Proteintech, 24790-1-AP; 1:1000), rabbit anti-4EHP (Gentex, GTX103977; 1:1000), rabbit anti-vinculin (Cell Signalling Technology, 13901S; 1:1000), rabbit anti-AGO2 (Cell Signalling Technology, 2897S; 1:1000) and mouse anti-cyclin D1 (Santa Cruz Biotechnology, sc-450; 1:1000). IRDye 800CW donkey anti-rabbit IgG (LI-COR, 926-32213) and IRDye 680RD donkey anti-mouse IgG (LI-COR, 926-68072) were used as secondary antibodies. All blots were scanned and images were taken using the Odyssey system (LI-COR, ODY-1540). ImageJ software was used for quantification of cyclin D1 protein expression. The density of cyclin D1 bands was measured and normalized to the density of the corresponding β-actin band as the loading control. The cyclin D1 densitometry in NSP2-expressing cells was compared to that in the GFP-expressing cells as control. The uncropped images of all blots are provided in Figs S4–S11.

### Co-immunoprecipitation

For co-immunoprecipitation assays, 4×10^6^ HEK293 cells were seeded in 10-cm plates and transfected the next day with 5 µg Flag–GFP or Flag–NSP2 plasmids using Lipofectamine 2000 according to the manufacturer's instructions. After 24 h, cells were washed with ice-cold PBS and collected by scraping in lysis buffer (40 mM HEPES pH 7.5, 120 mM NaCl, 1 mM EDTA, 0.3% CHAPS), supplemented with complete EDTA-free protease inhibitor tablet (Roche, 11836170001)]. After a 30 min incubation on ice with end-to-end rotation, the lysates were separated from debris by centrifugation at 14,000 ***g*** for 15 min at 4°C. The supernatants were pre-cleared by incubation with 50 µl washed and blocked Dynabeads Protein G (Thermo Fisher Scientific, 10004D) for 1 h at 4°C. 2 mg pre-cleared lysate was incubated with 3 µg anti-Flag antibody, 60 µl Dynabeads Protein G and 1 µl RNase I (Invitrogen, AM2294) with end-to-end rotation at 4°C overnight. Beads were washed three times for 10 min with wash buffer (50 mM HEPES pH 7.5, 150 mM NaCl, 1 mM EDTA, 0.3% CHAPS and complete EDTA-free protease inhibitor tablet). Protein was eluted in 2× SDS sample buffer for subsequent analysis by western blotting.

### Polysome profiling

For polysome profiling, 8×10^6^ HEK293 cells were seeded in 15 cm plates and transfected the next day with 15 µg Flag–GFP or Flag–NSP2 plasmid using Lipofectamine 2000 according to the manufacturer's instructions. After 24 h, cells were pretreated with cycloheximide (100 μg/ml; Sigma-Aldrich, 01810) for 5 min, collected by centrifugation at 1200 ***g*** for 5 min at 4°C and lysed in 500 μl hypotonic buffer [5 mM Tris-HCl pH 7.5, 2.5 mM MgCl_2_, 1.5 mM KCl, complete EDTA-free protease inhibitor tablet, 100 U Ribolock (Thermo Fisher Scientific, EO0382), 100 μg/ml cycloheximide, 2 mM dithiothreitol, 0.5% v/w Triton X-100 and 0.5% v/w sodium deoxycholate]. The lysates were cleared by centrifugation at 20,000 ***g*** for 5 min at 4°C. Total RNA concentration in the supernatant was measured using a NanoDrop 2000 (Thermo Fisher Scientific) at 254 nm, and the equivalent of 300 μg of RNA was diluted to a final volume of 500 μl and separated on 14 ml of a 10–50% sucrose gradient by ultracentrifugation at 230,500 ***g*** for 2 h in an SW40 rotor (Beckman Coulter) at 4°C. Absorbance at 254 nm was measured from lower to higher sucrose gradients using an ISCO gradient fractionation system and the optical density at 254 nm was continuously recorded with a Foxy JR Fractionator (Teledyne ISCO).

### Statistical analyses

Statistical tests were performed using Prism 6 (GraphPad). Error bars represent standard deviation (s.d.) from the mean of independent replicates. The numbers of replicates used in each analysis are indicated in the corresponding figure legends. *P*<0.05 were considered significant.

## Supplementary Material

10.1242/joces.261286_sup1 Supplementary information
