# Supplementary material for: The SARS-CoV-2 protein NSP2 enhances microRNA-mediated translational repression
Source: J Cell Sci. 2023 Oct 11;136(19):jcs261286. doi: 10.1242/jcs.261286 (PMC10617620; doi:10.1242/jcs.261286)
Supplement: Supplementary information [file joces-136-261286-s1.pdf]

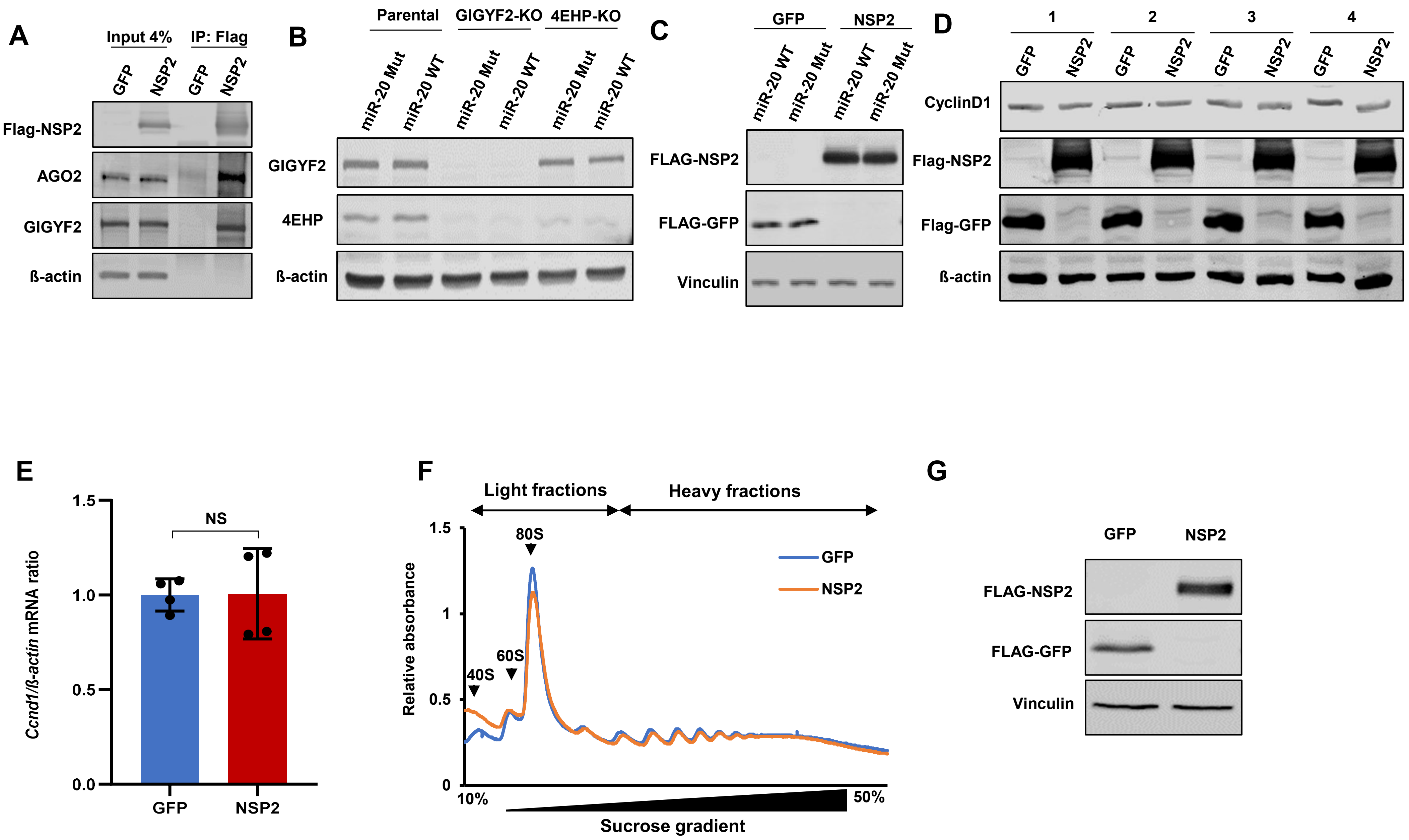

Fig. S1. Western blot analysis of HEK293 cell lysates; Related to Figure 1. (**A**) HEK293 cells were transfected with Flag-GFP or Flag-NSP2 plasmid. 24 h later, cell lysates were immunoprecipitated using anti-Flag antibody and western blot was performed with the specified antibodies. (**B**) Western blot analysis of lysates from parental, GIGYF2- KO, and 4EHP-KO HEK293 cells transfected with *FL*-miR-20 WT or *FL*-miR-20 Mut with the indicated antibodies. (**C**) Western blot analysis of lysates from HEK293 cells transfected with *FL*-miR-20 WT or *FL*-miR-20 Mut along with Flag-GFP or Flag-NSP2 with the indicated antibodies. (**D**) Western blot analysis of the expression of Cyclin D1 protein in lysates derived

from HEK293 cells that express Flag-NSP2 or Flag-GFP as control (4 independent replicates). ß-actin was used as loading control. (**E**) RT-qPCR analysis of *Ccnd1* mRNA relative to *ß-actin* expression in Flag-GFP or Flag-NSP2 expressing HEK293 cells, 24 h after transfection. Data are presented as mean ± SD (n=3). NS=non-significant (two-tailed Student's t-test). (**F**) Polysome profiling using Flag-GFP or Flag-NSP expressing HEK293 cells. (**G**) Western blot analysis of lysates from HEK293 cell described in F. Vinculin was used as loading control.

Journal of Cell Science • Supplementary information

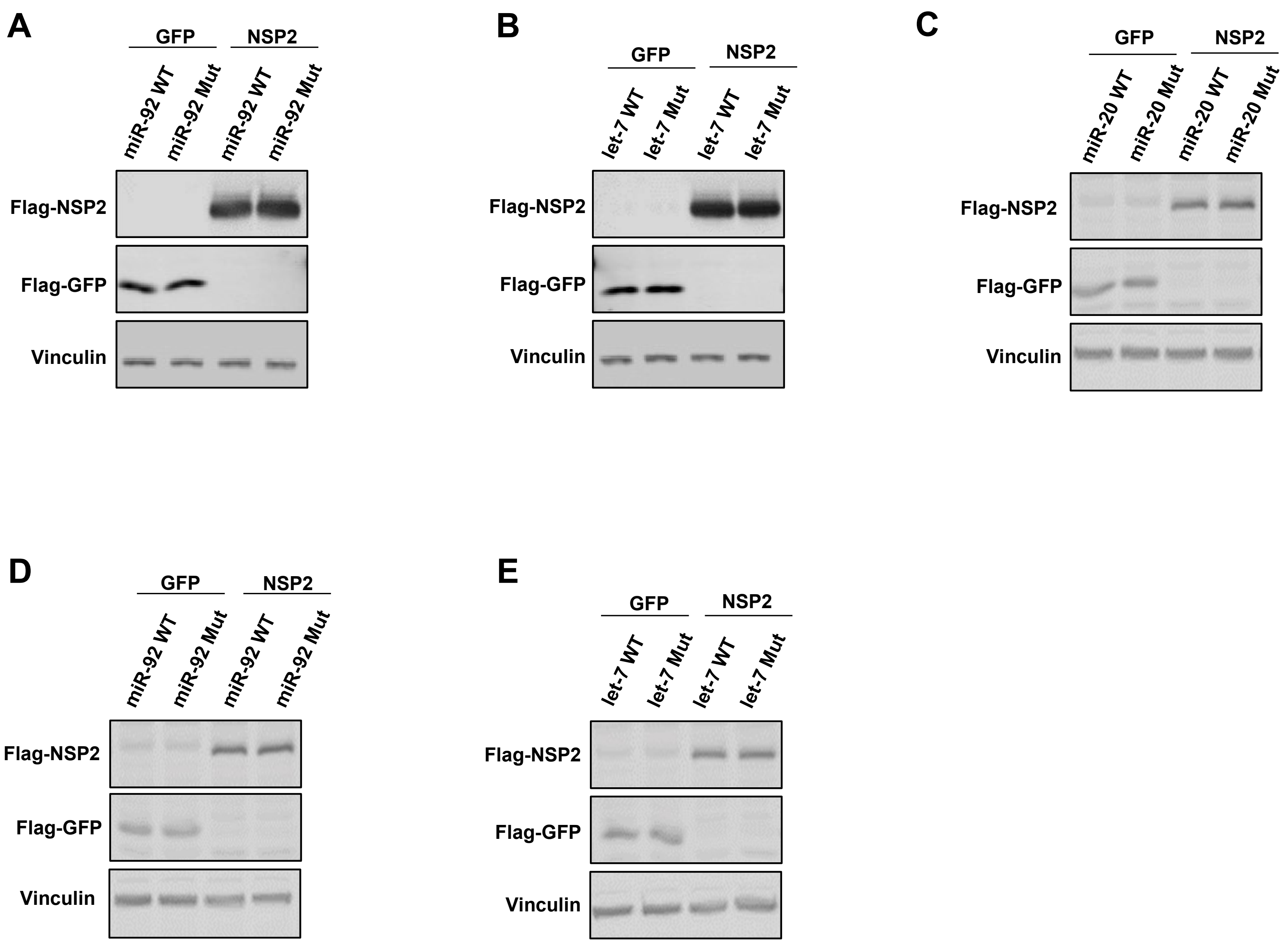

Fig. S2. Western blot analysis of HEK293 and U87 cell lysates; Related to Figure 2. (**A** & **B**) Western blot analysis of lysates from HEK293 cells transfected with *FL*- miR-92 WT or *FL*-miR-92 Mut (**A**) or *FL*-let-7 WT or *FL*-let-7 Mut (**B**) in the presence of Flag- NSP2 or Flag-GFP with the indicated antibodies. (**C-E**) Western blot analysis of lysates from U87 cells transfected with *FL*-miR-20 WT or *FL*-miR-20 Mut (**C**) *FL*-miR-92 WT or *FL*-miR-92 Mut (**D**), *FL*-let-7 WT or *FL*-let-7 Mut (**E**) in the presence of Flag-NSP2 or Flag-GFP with the indicated antibodies.

Journal of Cell Science • Supplementary information

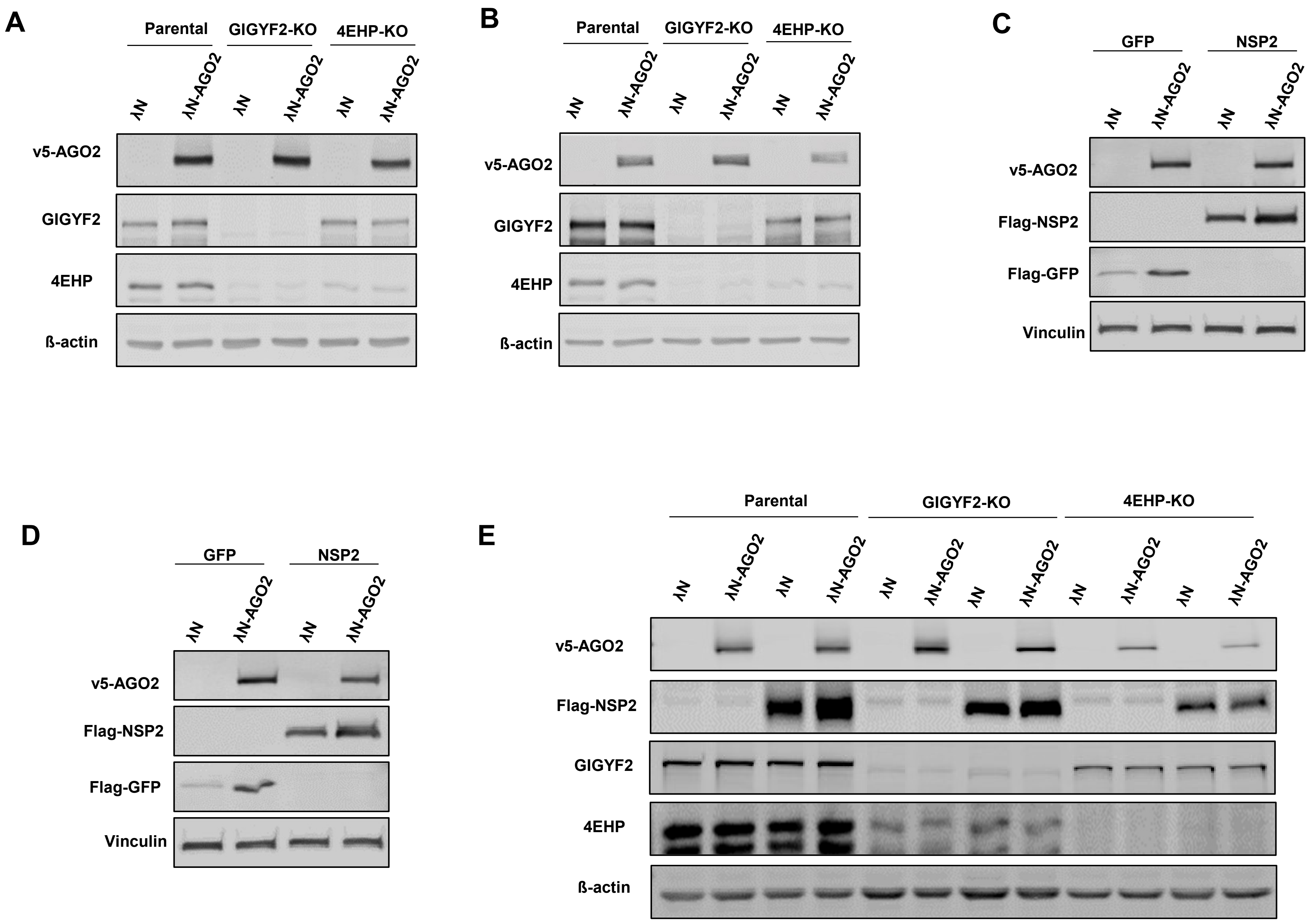

Fig. S3. Western blot analysis of HEK293 cell lysates; Related to Figure 4. (**A**) Western blot with the indicated antibodies using lysates from the parental, GIGYF2-KO, and 4EHP-KO HEK293 cells transfected with *RL*-5BoxB reporter and λN-AGO2 or λN-empty plasmids. (**B**) Western blot with the indicated antibodies using lysates from the parental, GIGYF2-KO, and 4EHP-KO HEK293 cells transfected with *RL*-5BoxB-HhR reporter and λN-AGO2 or λN-empty plasmids. (**C**) Western blot with the indicated antibodies using lysates from the HEK293 cells transfected with *RL*-5BoxB reporter and λN-AGO2 or λN-empty plasmids, along with Flag-NSP2 or Flag-GFP. (**D**) Western blot with the indicated antibodies 33 λN-empty plasmids, along with Flag-NSP2 or Flag-GFP. (**E**) Western blot with the indicated antibodies using lysates from the GIGYF2-KO, 4EHP-KO and parental HEK293 cells transfected with *RL*-5BoxB-HhR reporter and λN-AGO2 or λN-empty plasmids, along with Flag-NSP2 or Flag-GFP.

Journal of Cell Science • Supplementary information

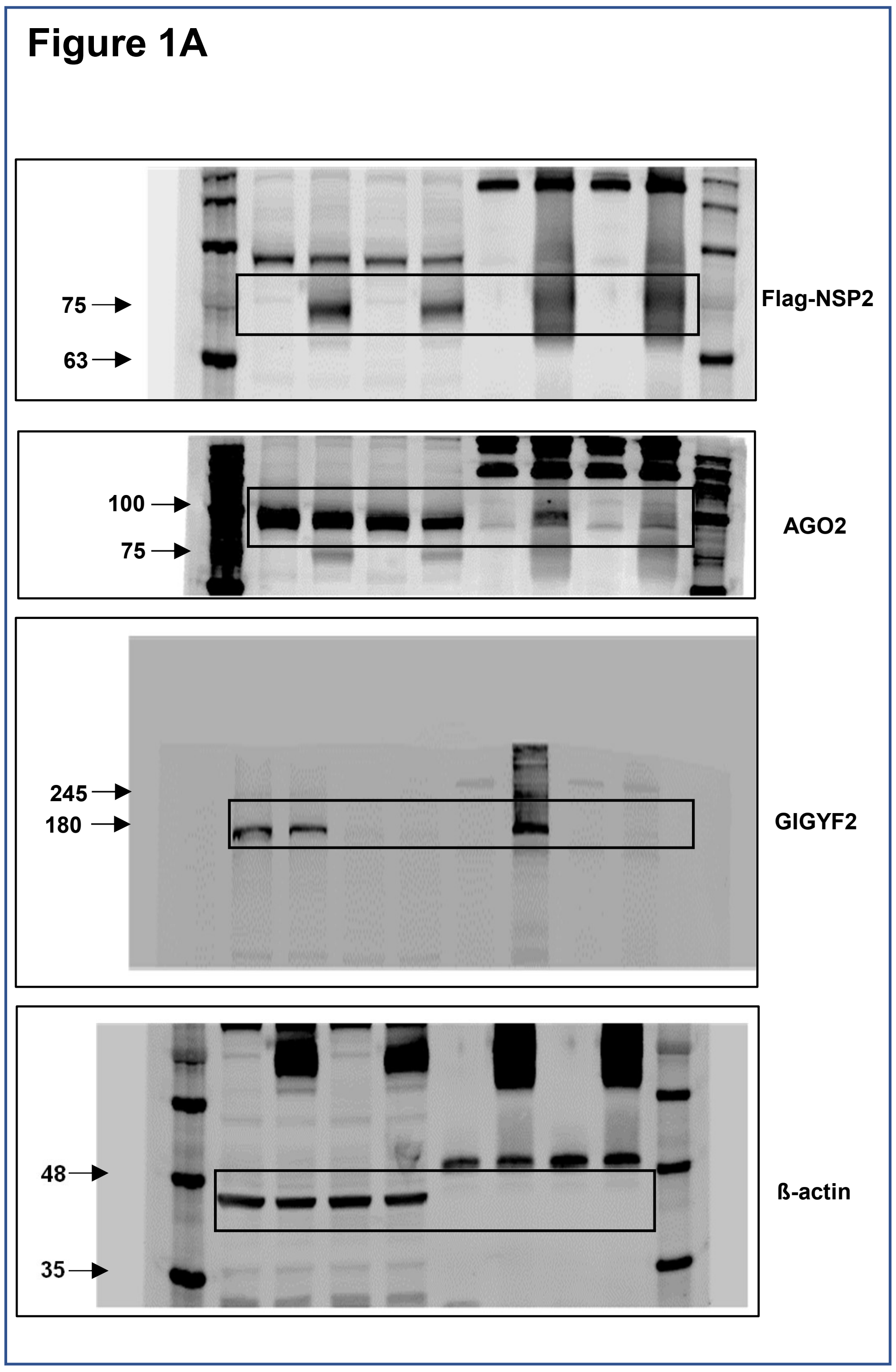

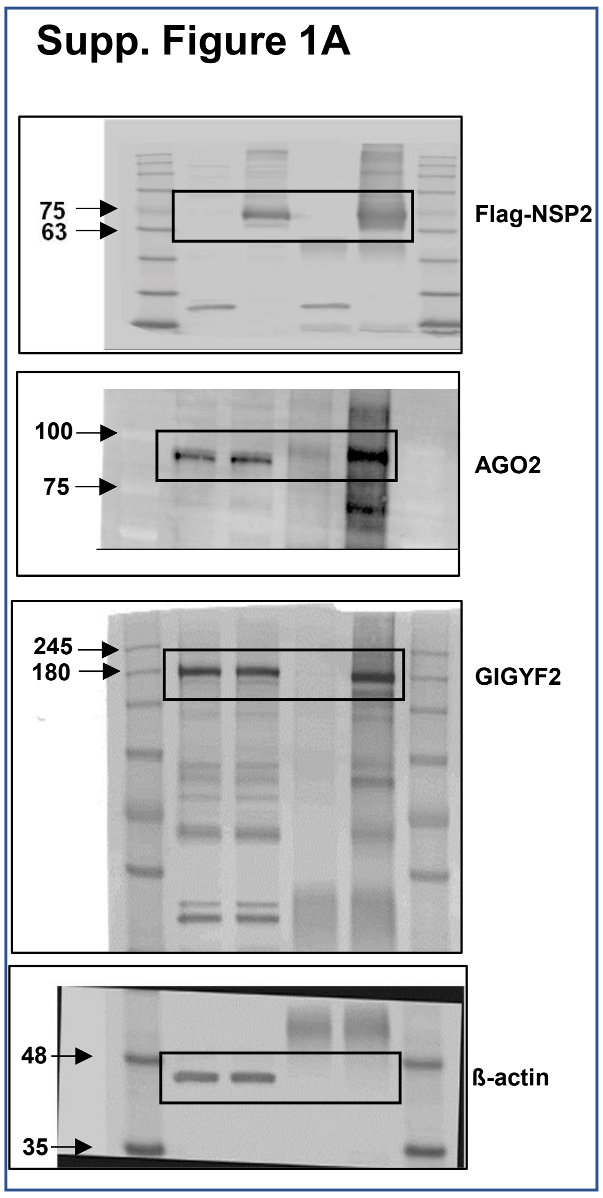

Fig. S4. Uncropped images of blots used in Figures 1A and Supp. Figure 1A.

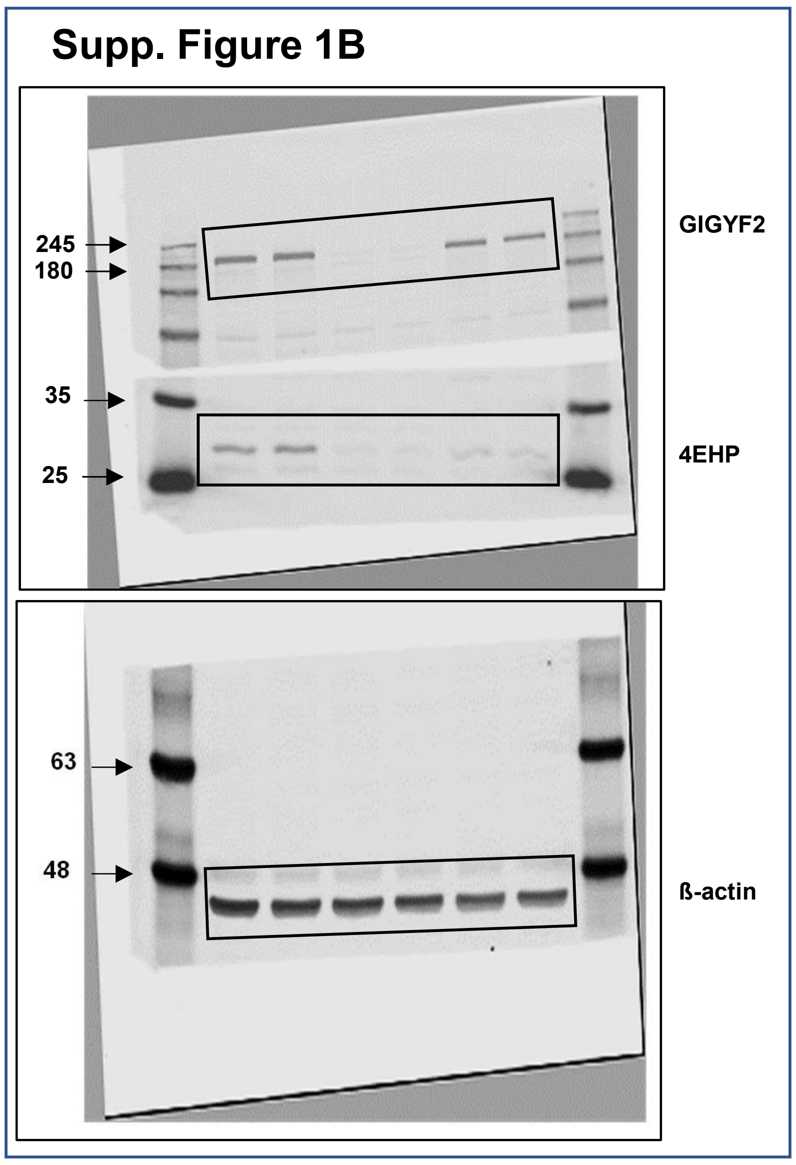

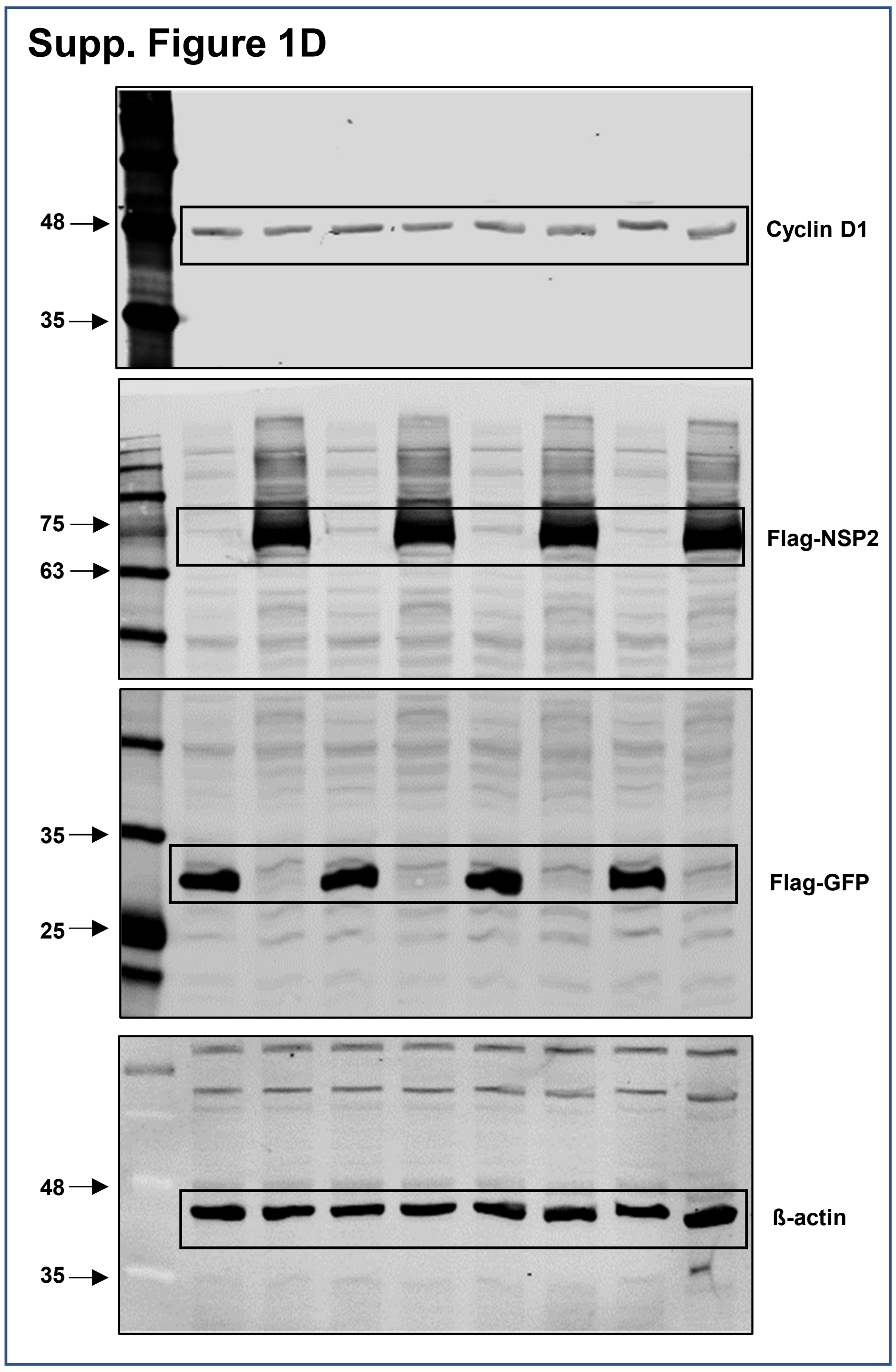

Fig. S5. Uncropped images of blots used in Supp. Figure 1B, 1D.

Journal of Cell Science • Supplementary information

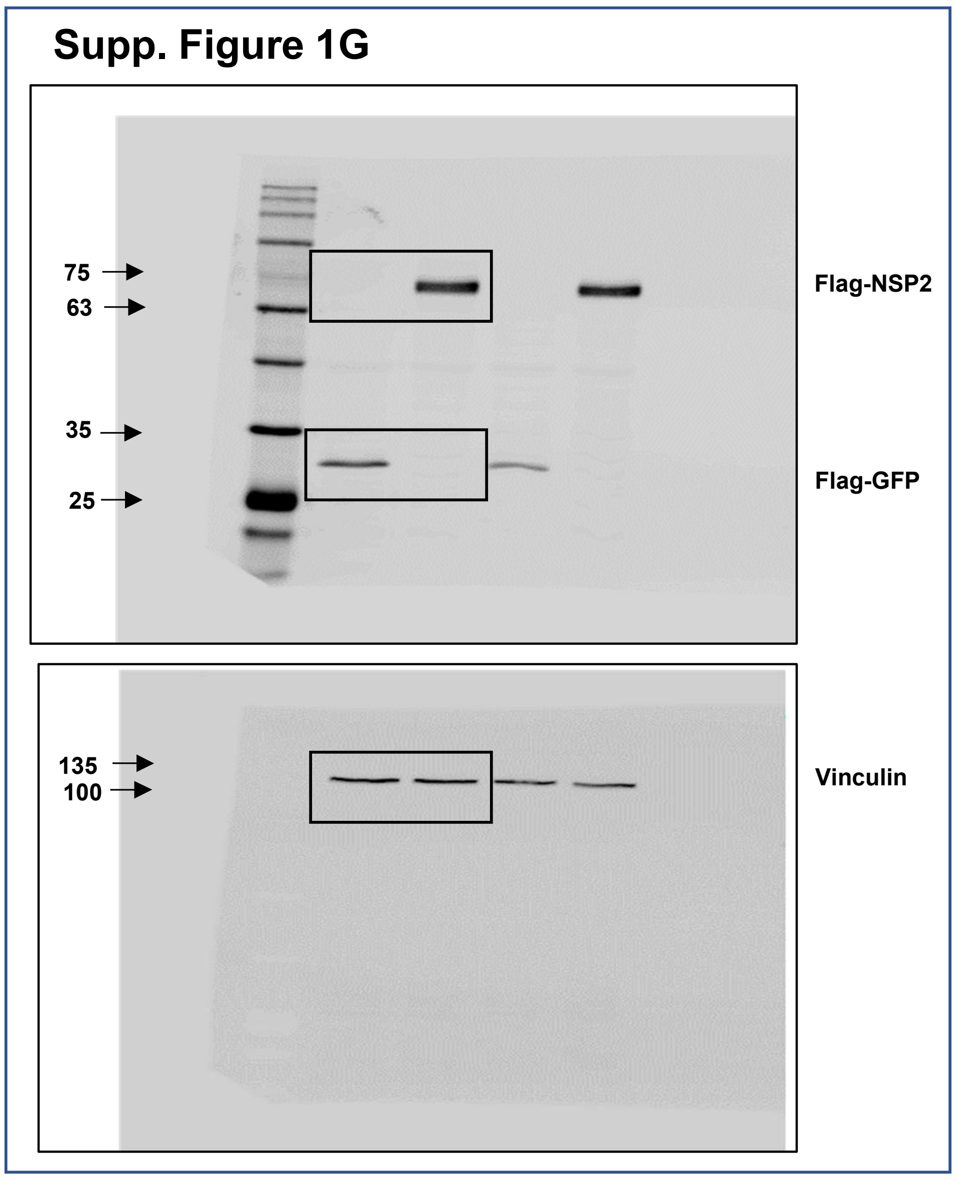

Fig. S6. Uncropped images of blots used in Supp. Figure 1G.

Journal of Cell Science • Supplementary information

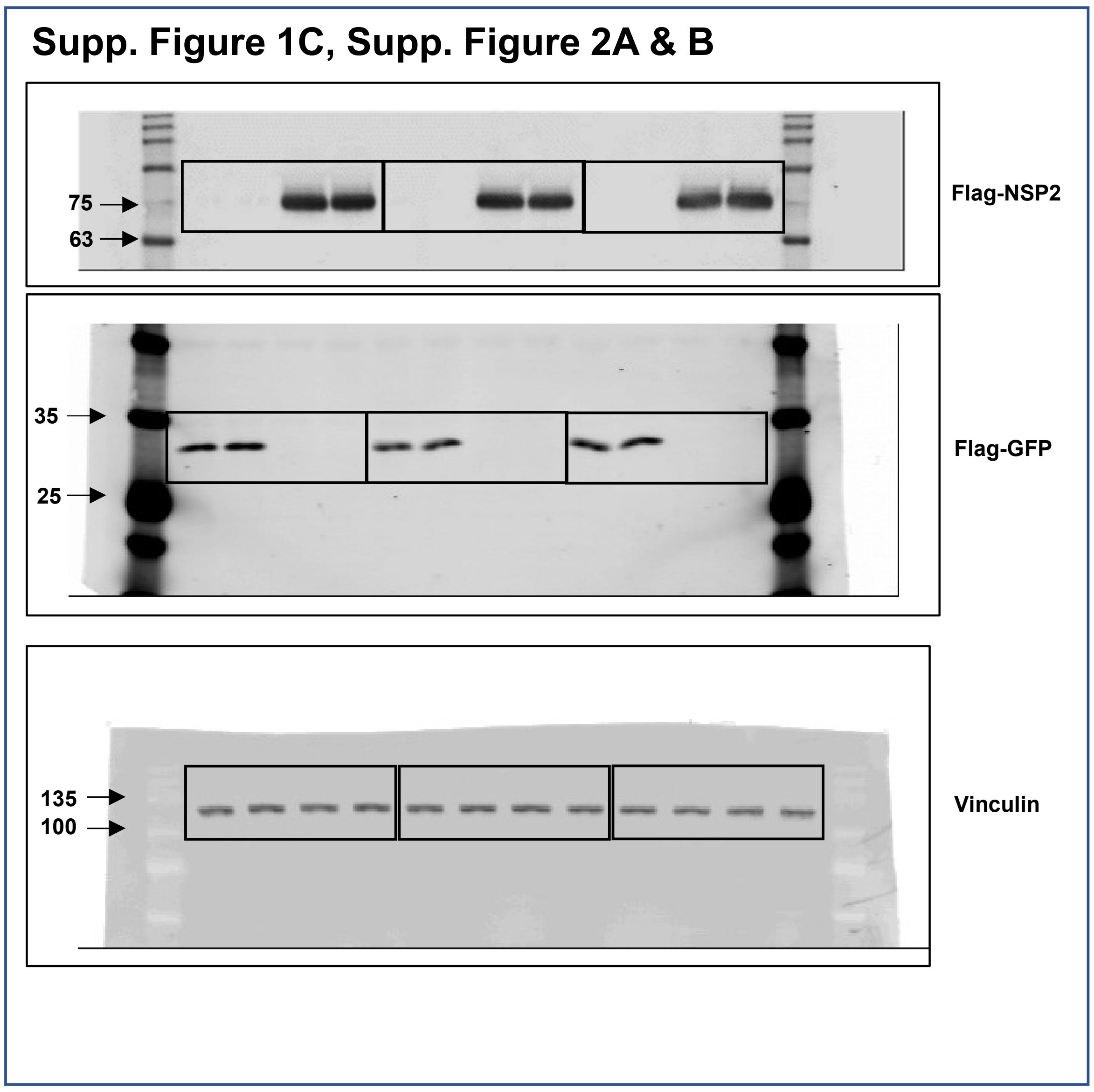

Fig. S7. Uncropped images of blots used in Supp. Figure 1C, Supp. Figure 2A & B.

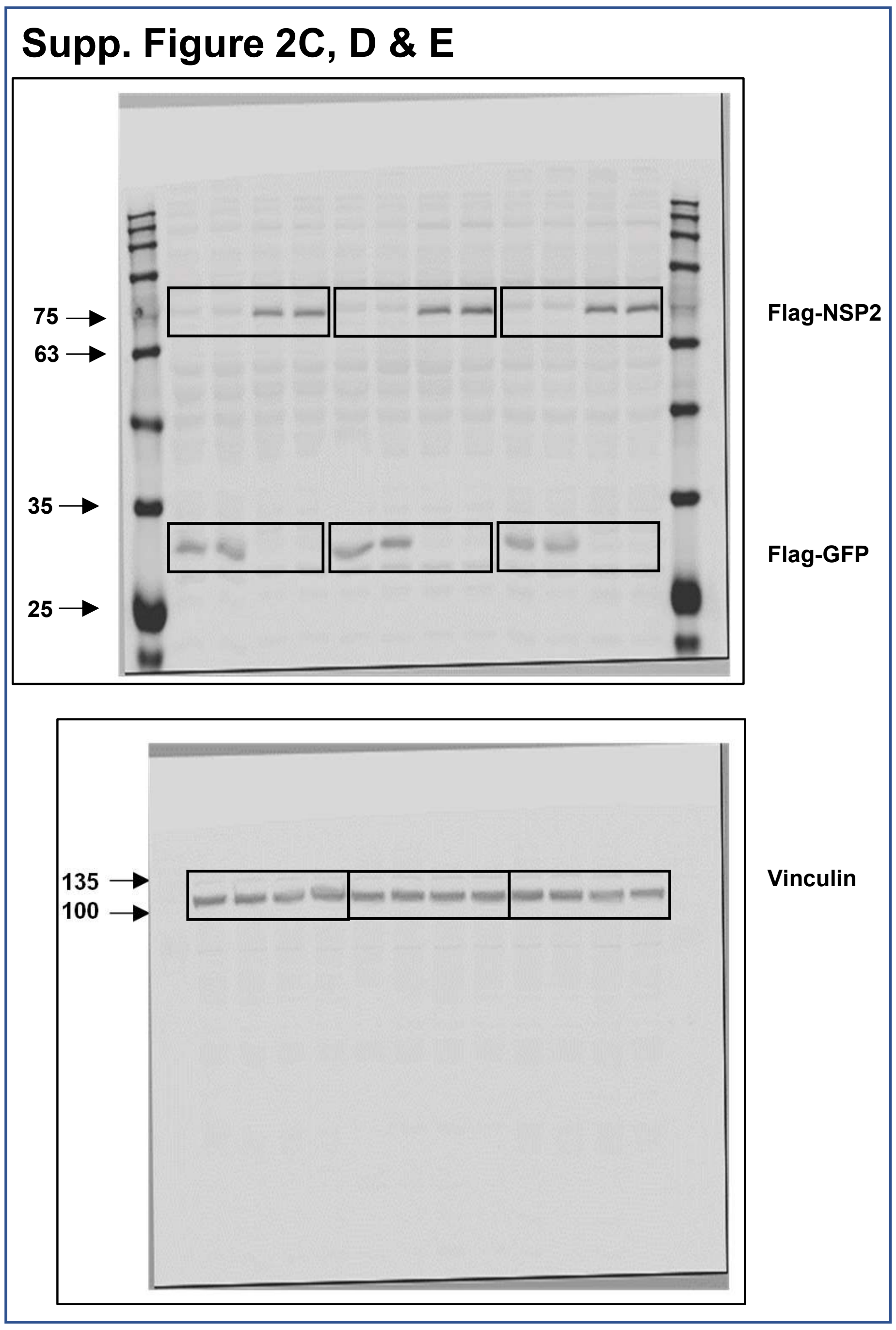

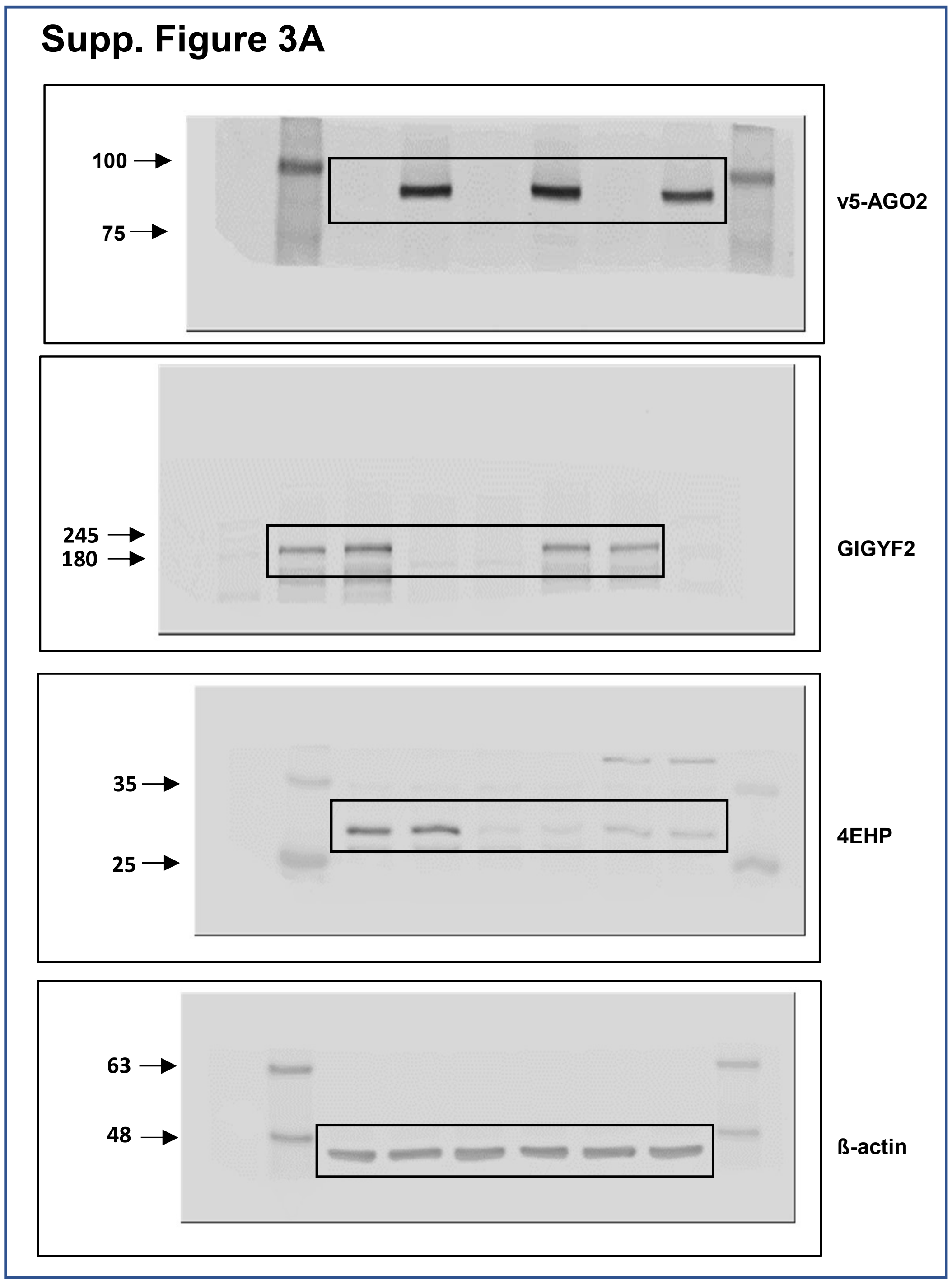

Fig. S8. Uncropped images of blots used in Supp. Figure 2C-E, and Supplementary Figure 3A.

Journal of Cell Science • Supplementary information

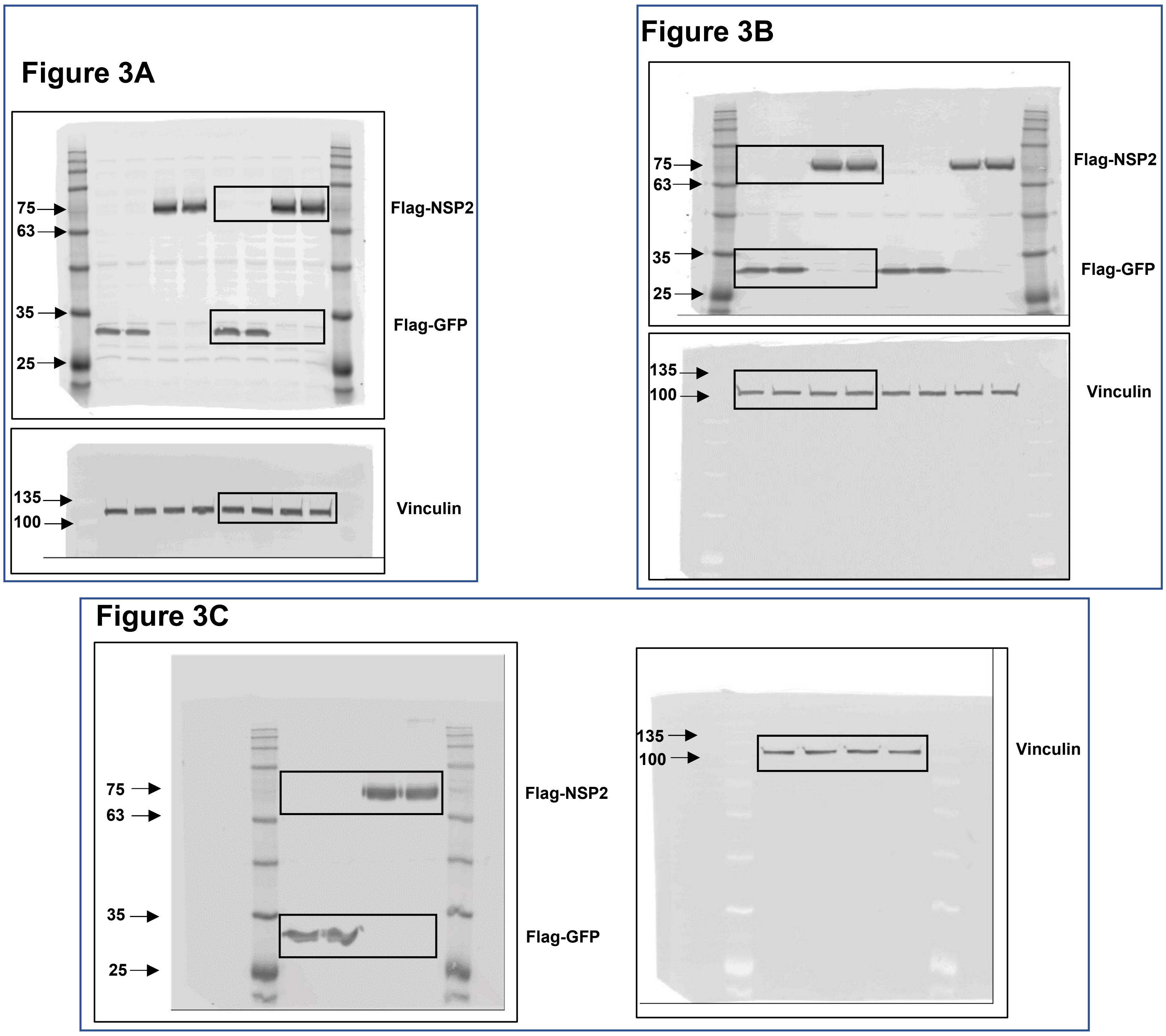

Fig. S9. Uncropped images of blots used in Figure 3A-C.

Journal of Cell Science • Supplementary information

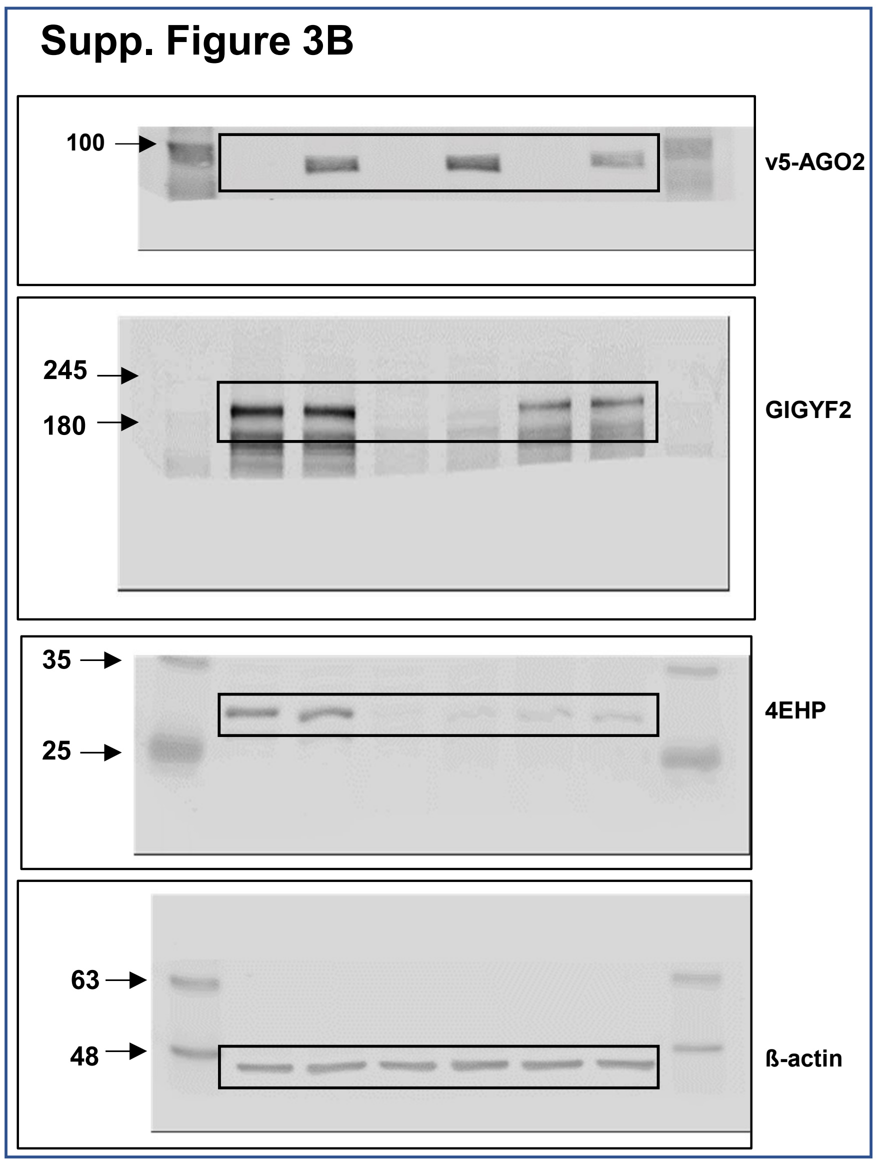

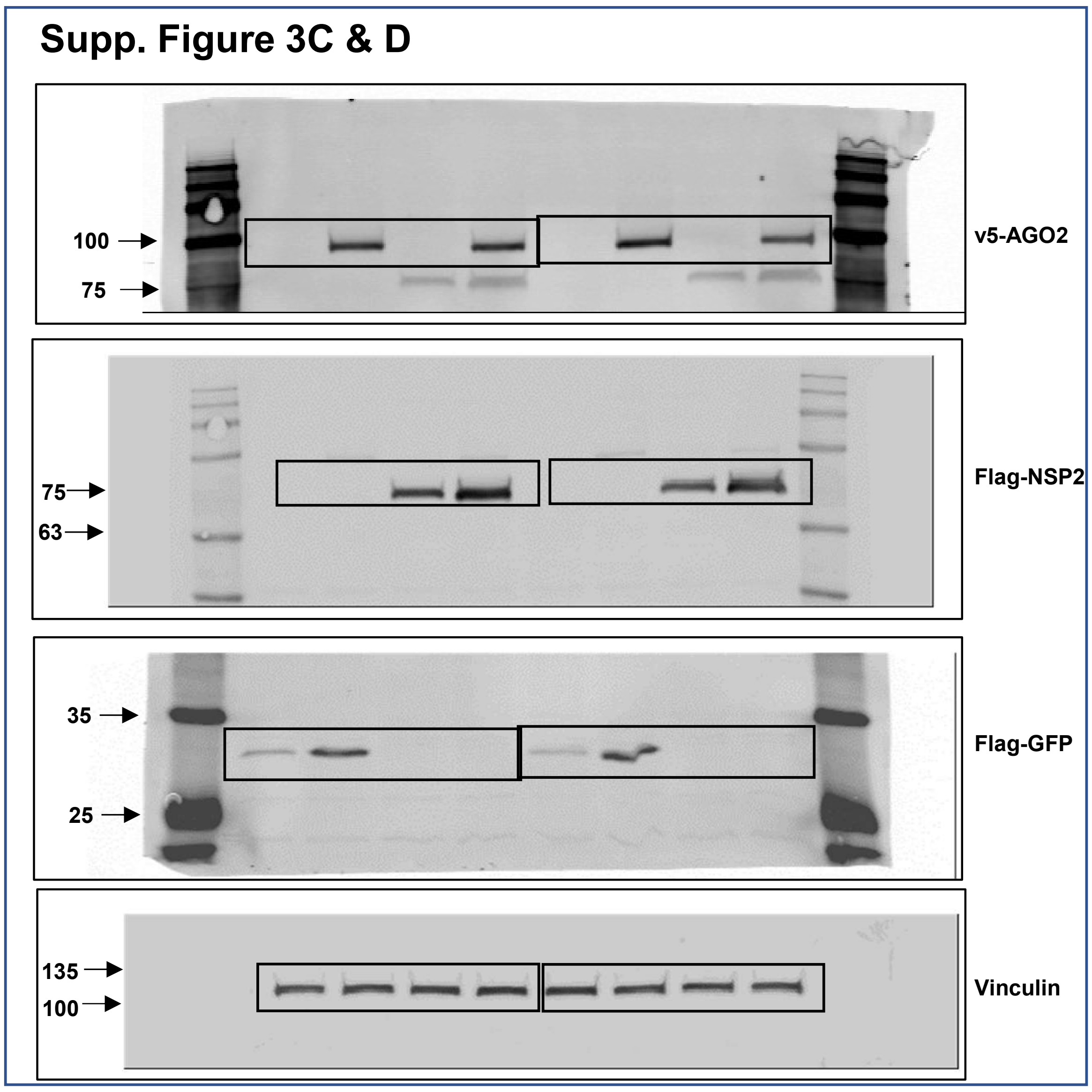

Fig. S10. Uncropped images of blots used in Supp. Figure 3B-D.

Journal of Cell Science • Supplementary information

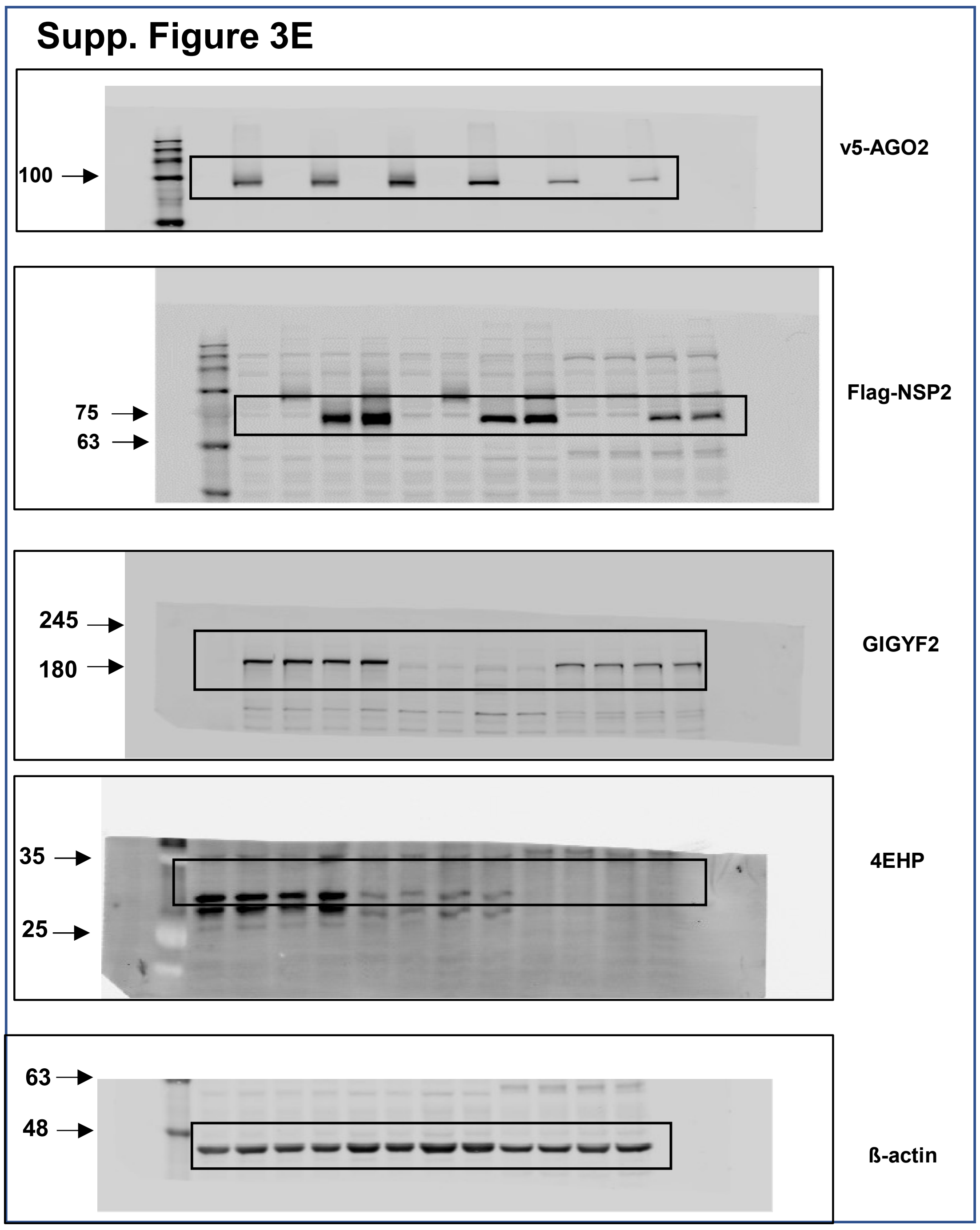

Fig. S11. Uncropped images of blots used in Supp. Figure 3E.

Journal of Cell Science • Supplementary information
