# Supplementary material for: The SARS-CoV-2 protein NSP2 enhances microRNA-mediated translational repression
Source: J Cell Sci. 2023 Oct 11;136(19):jcs261286. doi: 10.1242/jcs.261286 (PMC10617620; doi:10.1242/jcs.261286)
Supplement: Supplementary file 1 [file joces-136-261286_review_history.pdf]

The SARS-CoV-2 protein NSP2 enhances microRNA-mediated translational repression

Parisa Naeli, Xu Zhang, Patric Harris Snell, Susanta Chatterjee, Muhammad Kamran, Reese Jalal Ladak, Nick Orr, Thomas Duchaine, Nahum Sonenberg and Seyed Mehdi Jafarnejad

DOI: 10.1242/jcs.261286

Editor: Maria Carmo-Fonseca

Review timeline
Submission to Review Commons: 31 December 2022
Original submission: 26 April 2023
Editorial decision: 28 April 2023
First revision received: 2 September 2023
Accepted: 8 September

Reviewer 1

Evidence, reproducibility and clarity

In this manuscript the authors examine whether SARS-CoV-2 protein, NSP2 mediate translation repression of cellular transcripts by increasing miRNA mediated suppression. The same authors previously demonstrated NSP2 interacts with GIGYF2 and this interaction suppresses the translation of the Ifnb mRNA. Here they extend this finding and using a series of reporters they illustrate that at least part of NSP2 translation suppression is mediated by increasing GIGYF2 mediated miRNA translation suppression.
Overall the manuscript is clearly written and the experiments well executed.

Major comments:

Optional- The authors show that the ability of NSP2 to enhance miRNA-mediated repression is dependent on the extent of the initial repression and suggest this dependency on the initial repression levels may explain the discrepancy with published work showing NSP2 actually impairs (and not increase) microRNA-mediated silencing . Therefore, an important addition could be to examine what happen to translation in cells that express NSP2 and whether generally translation repression is significant in transcripts (native) that are enriched in miRNA binding site. This experiment will help to show NSP2 effect on native transcripts and potentially help to understand how much of these changes are likely explained by miRNAs.

Minor comments:

1. Personally I found the term repression fold quite confusing and unintuitive. Why not to present the actual measurements so that the control (blue bars) have high value and the red bars (representing repression) have lower values?
It seems inadequate to present on the same graph two values that are normalized to 1. For example, in figure 1D it will be important to show the mir20-Mut real values (at least mir20-Mut in NSP2 cells should not be normalized to 1). This will allow to show that the differences are indeed mediated by stronger translation repression of miR-20 WT luciferase in the presence of NSP2 and not by unexplained differences in mir20-mut luciferase expression. This is true for almost all the figures in the manuscript. Correspondingly, the statistical test should be two-factor ANOVA test examining if NSP2 expression significantly increase the difference between Luciferase miR20-WT and luciferase miR20-mut.

© 2023. Published by The Company of Biologists under the terms of the Creative Commons Attribution License (https://creativecommons.org/licenses/by/4.0/).

Significance

There is an urgent need for better molecular understanding of how SARS-CoV-2 proteins influence the machineries of the host cell.

This study investigates how NSP2 interaction with GIGYF2 mediate translation repression of cellular transcript. The authors also address the discrepancy with previously published work that showed NSP2 actually impairs (and not increase) microRNA-mediated silencing.

The paper would be of interest to RNA biologists and for molecular virologists that study SARS-CoV-2

Reviewer 2

Evidence, reproducibility and clarity

In this study, Naeli and collaborators propose a role for the SARS-CoV2 protein NSP2 in the miRNA-mediated translational repression. Their data show that NSP2 co-immunoprecipitate with the Argonaute AGO2 and the previously reported translation modulator GIGYF2. Using different reporters sensitive to miRNA repression, they show that overexpressing NSP2 enhances miRNA-mediated translational repression in different cell lines as well as one endogenous miRNA target CDK1 and thus without affecting the stability of targeted mRNAs. Interestingly, their data show that the effect of NSP2 depends on the number of miRNA binding sites suggesting that highly repressed mRNAs, due to the presence of several miRNA binding sites, are not affected by NSP2. Finally, using an AGO2 tethering reporter assay that does not rely on miRNA binding but needs two NSP2 interactors GIGYF2 and 4EHP, they demonstrate that overexpressing NSP2 still stimulates mRNA repression, suggesting that NSP2 can have a broader impact on miRISC-dependent silencing.

Overall, this is an interesting follow-up study from their previous paper (Xu et al., 2022) that have the potential to add another dimension to the modulation of mRNA translation by the SARS-CoV2 protein NSP2. But unfortunately, in its current form, the work falls short of supporting their claims appropriately and providing sufficient and relevant insights about the role of NSP2 in regulating the miRNA-mediate gene regulation. To overcome this, the authors should perform and add the following experiments to strengthen their study.

1. Interaction of NSP2 with the miRISC: With the data presented here, we can only conclude that NPS2 forms a complex with AGO2. Is their interaction direct or indirect? Is miRNA- and TNRC6-bound AGO2 (and thus miRISC) associated with NSP2? To address those important questions, the authors should perform NSP2 immunoprecipitations in GIGYF2 (and 4EHP) KO cell lines and monitor the presence of AGO2 and miRNAs in the immunoprecipitated complex. Those experiments will define the type of interaction NSP2 has with the miRISC (GIGYF2 dependent or not).
2. Along this line, is NSP2 action on miRNA-mediated gene regulation dependent or not of GIGYF2? Again, testing this by monitoring the repression of the different reporters upon NSP2 overexpression in the presence or not of GIGYF2 in cells will precise the contribution of NSP2 in miRNA-mediated gene silencing.

Besides these two sets of critical experiments that will define the relationship between NSP2 mRNA modulation and the microRNA pathway, it would be interesting for this study to readily test the effect of NSP2 on miRNA targets related to immune-regulatory processes and antiviral response. As the authors pointed out in their discussion, several mRNAs are involved in the control of genes found in those pathways. Demonstrating the contribution of NSP2 in the regulation of a few of them will strengthen their study's significance and interest by providing a possible mechanism at play during a SARS-CoV2 infection. Also, it would be interesting to test if the modulation of translation inhibition by NSP2 occurs in cells infected by the SARS-CoV2 virus. For instance, is NSP2 and miRISC interaction enhances during the viral infection? As the authors propose, NSP2 could also have improved antiviral microRNA repression, so it would be interesting to characterize this interplay in a relevant biological context.

© 2023. Published by The Company of Biologists under the terms of the Creative Commons Attribution License (https://creativecommons.org/licenses/by/4.0/).

Minor comment:

From the data presented in Figure S1A, it is impossible to conclude "that miR-20a represses the expression of the target mRNA in a GIGFY2-dependent manner" as its KO also affect the level/stability of 4EHP. Therefore, we cannot distinguish the contribution of GIGFY2 and 4EHP in this context as both protein levels decrease. The authors should tone down this statement on page 3.

Significance

With the addition of the proposed experiments, the revised study will better define the direct contribution of NSP2 with the miRISC. This work has the potential to provide another aspect of the mRNA translation modulation by the SARS-CoV2 protein NSP2 with the interesting angle of miRNA-mediated gene silencing.

As mentioned by the authors, another recent paper reports the potential impact of NSP2 on post-transcriptional silencing (Zou et al., iScience 2022). However, in contrast to the current study, this previous work did not directly test the interaction of NSP2 with the miRISC. Furthermore, it only used a single miRNA reporter (let-7) to support NSP2 contribution in miRNA-mediated gene silencing, which does not demonstrate the broader impact of NSP2 on this gene regulatory mechanism as tested in this current study. Upon revision, this study will provide more definitive proof of the involvement of NSP2 in miRNA-mediated gene regulation and thus will be of interest to experts in the miRNA field and scientists interested in understanding viruses/hosts interaction.

Although not essential, if the authors want to add data that addresses the function of NSP2 on this regulatory pathway in the context of viral infection (which seems feasible for this group), that will definitely increase the broader significance of their work.

I am an expert in molecular biology and molecular genetics, miRNA-mediated gene regulation, and small non-coding RNA biology.

Reviewer 3

Evidence, reproducibility and clarity

The manuscript by Naeli et al., presents study on effects of SARS-CoV-2 NSP2 protein on miRNA mediated translational repression. Authors show in multiple ways using reporter mRNAs with various miRNA sites that NSP2 protein stimulates miRNA mediated repression. The increase in miRNA repression is likely due to the interaction of NSP2 with either GIGYF2 or Argonaute protein directly thus making more stable repressive complex on mRNA. The manuscript is written clearly and methods provide enough details for reproducibility. Only major comment would be that authors could have tested multiple endogenous targets of miRNAs for extent of miRNA mediated expression in the presence of NSP2. This could be achieved using either western blots for known targets (looking at protein levels) or using targeted qRT-PCR or distribution of mRNAs in polysome fractions (mRNA translational repression. An alternative would be Ribo-Seq experiment.

Minor comment:

Line in introduction arguing: "The GIGYF2/4EHP complex is recruited by a variety of factors including miRNAs" should state miRISC instead of miRNAs.

Significance

General assessment: The study presents sold evidence that NSP2 protein interacts with miRISC complex and increases miRNA-mediated translational repression of reporter mRNAs. Multiple target sites for miR20, let7 and miR92 are tested as well as two different human cell lines (Hek293 and U87) which gives strength to study and reproducibility of the results. Focus on the endogenous miRNA targets in the presence or absence of the NSP2 protein would make study even stronger.

© 2023. Published by The Company of Biologists under the terms of the Creative Commons Attribution License (https://creativecommons.org/licenses/by/4.0/).

The advance of the study is more rigorous analyses of NSP2 protein effects on miRNA- mediated gene expression regulation with some novel mechanistic insights. The study will be of interest for specialized and basic research audience with potential impact on translational research.

My field of expertise covers mechanisms of gene expression regulation by miRNAs and RBPs as well as impact of mRNAs, nascent peptides and ribosomes on protein synthesis.

Author response to reviewers' comments

We thank the reviewers for their positive assessments and providing constructive comments to greatly improve our manuscript. Following reviewers' suggestions, we performed new experiments and analyses, revised the text, and modified/added new figures to further support our conclusions. In addition, we generated a plan that involves performing additional experiments to address the remaining comments.

Reviewer #1
(Evidence, reproducibility and clarity (Required)):
In this manuscript the authors examine whether SARS-CoV-2 protein, NSP2 mediate translation repression of cellular transcripts by increasing miRNA mediated suppression. The same authors previously demonstrated NSP2 interacts with GIGYF2 and this interaction suppresses the translation of the Ifnb mRNA. Here they extend this finding and using a series of reporters they illustrate that at least part of NSP2 translation suppression is mediated by increasing GIGYF2 mediated miRNA translation suppression.
Overall the manuscript is clearly written and the experiments well executed.

Major comments:
Optional- The authors show that the ability of NSP2 to enhance miRNA-mediated repression is dependent on the extent of the initial repression and suggest this dependency on the initial repression levels may explain the discrepancy with published work showing NSP2 actually impairs (and not increase) microRNA-mediated silencing. Therefore, an important addition could be to examine what happen to translation in cells that express NSP2 and whether generally translation repression is significant in transcripts (native) that are enriched in miRNA binding site. This experiment will help to show NSP2 effect on native transcripts and potentially help to understand how much of these changes are likely explained by miRNAs.

Response: We thank the reviewer for this suggestion. To analyse the changes in general mRNA translation by NSP2, we performed polysome profiling assay in HEK293 cells transfected with NSP2 or GFP as a control. We observed that NSP2 overexpression does not have an impact on the general mRNA translation in the cells (Rebuttal Fig. 1 & Fig. S1E in the revised manuscript). Importantly, this observation is expected. Our previous analyses of the changes in mRNA translation upon depletion of GIGYF2 or 4EHP, the two cellular factors that effect the microRNA-mediated translational repression and form a complex with NSP2, had also shown no apparent change in general mRNA translation (Jafarnejad, et al, eLife 2018 & Zhang, et al, Molecular Cell 2021)[1, 2]. This is likely because these factors affect mRNA translation in a transcript-specific manner.

In our planned revision of this manuscript, we will analyse the impact of NSP2 on translation of known native microRNA target transcripts, including *Hmga2* & *Ccnd1*, which are known microRNA target mRNAs and our reporter assay (Fig. 3C) and western blot (Fig. 1F) analyses indicates their likely sensitivity to NSP2-induced translational repression. We will assess the impact of NSP2 on translation of these native transcripts by analysing their distribution in sucrose gradients.

© 2023. Published by The Company of Biologists under the terms of the Creative Commons Attribution License (https://creativecommons.org/licenses/by/4.0/).

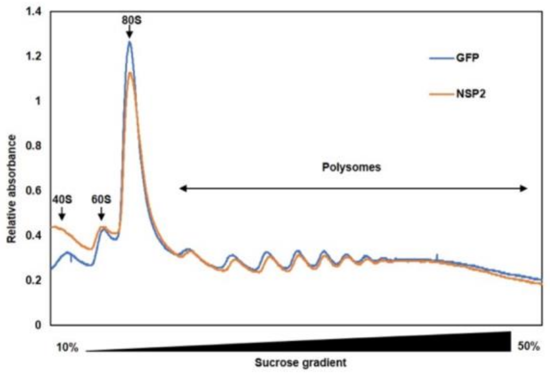

Rebuttal Figure 1: Polysome profiling assay in HEK293 cells 24 h after transfection with NSP2 or GFP control.

Minor comments:

1. Personally I found the term repression fold quite confusing and unintuitive. Why not to present the actual measurements so that the control (blue bars) have high value and the red bars (representing repression) have lower values?

Response: "Repression fold" has been used by us[3] and others[4] in the literature relevant to regulation of mRNA translation due to its effectiveness in visual depiction of relatively smaller changes in repression of mRNA translation, compared with the alternative "expression ratio". For instance, we have compared the two methods of visualisation of the same data (increased repression of Let-7 reporter in HEK293 cells by NSP2; Rebuttal Fig. 2), based on which we believe the "Repression fold" method is better in depicting even small changes in repression of the reporter mRNA.

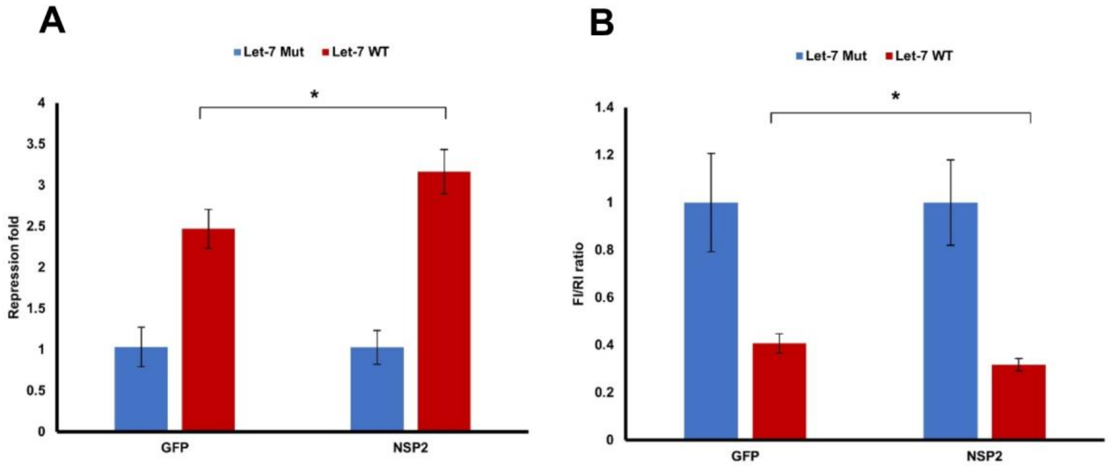

Rebuttal Figure 2. NSP2-induced enhanced repression mediated by let-7 depicted using repression fold (**A**) and expression ratio (**B**) methods.

2. It seems inadequate to present on the same graph two values that are normalized to 1. For example, in figure 1D it will be important to show the mir20-Mut real values (at least mir20-Mut in NSP2 cells should not be normalized to 1). This will allow to show that the differences are indeed mediated by stronger translation repression of miR-20 WT luciferase in the presence of NSP2 and not by unexplained differences in mir20-mut luciferase expression. This is true for almost all the figures in the manuscript. Correspondingly, the statistical test should be two-factor ANOVA test examining if NSP2 expression significantly increase the difference between Luciferase miR20-WT and luciferase miR20-mut.

Response: We have used normalization of mutant reporter for this assay, along with two-tailed Student t-test statistical analysis, as it is the common practice in the field[5-9]. In order to compare

© 2023. Published by The Company of Biologists under the terms of the Creative Commons Attribution License (https://creativecommons.org/licenses/by/4.0/).

the outcome between the current method and the one suggested by the reviewer, we performed parallel analysis of 3 experiments with normalized mutant reporter and paired student t-test (Rebuttal Fig. 3 A, C, & E) vs non-normalized mutant reporter and 2-way ANOVA test (Rebuttal Fig. 3B, D, & F), which revealed very similar outcomes (statistically significant NSP2-induced enhanced translational repression induced by miRNAs), regardless of the type of analysis.

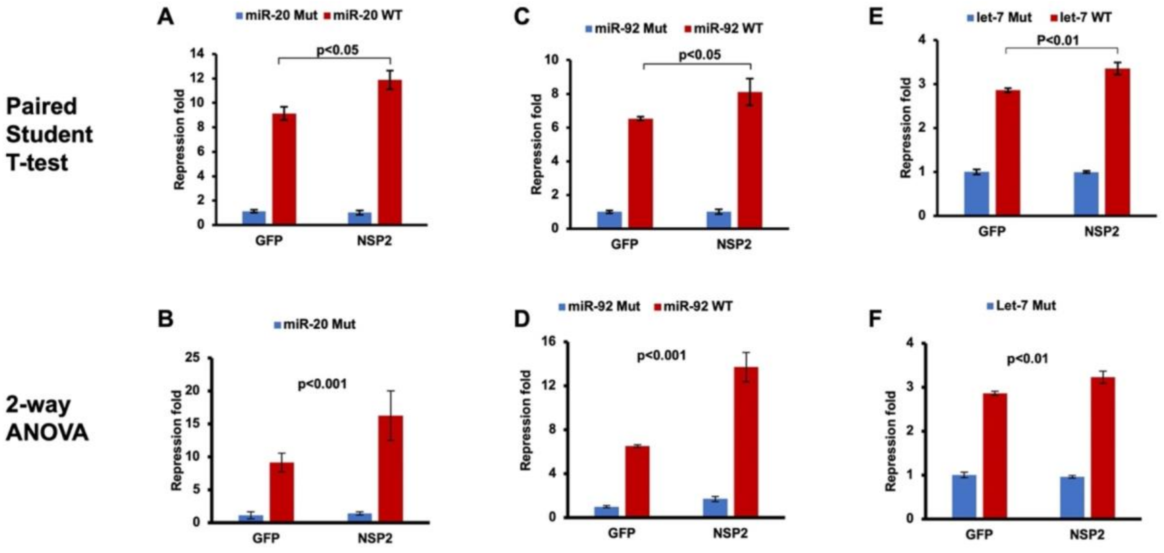

Rebuttal Figure 3. Analysis of the effect of NSP2 on repression induced by miR-20, miR-92, and Let-7 miRNAs in HEK293 cells using paired student t-test (Panels A, C, & E) vs non-normalized mutant reporter and 2-way ANOVA test (Panels B, D, & F).

Reviewer #1 (Significance (Required)):
There is an urgent need for better molecular understanding of how SARS-CoV-2 proteins influence the machineries of the host cell. This study investigates how NSP2 interaction with GIGYF2 mediate translation repression of cellular transcript. The authors also address the discrepancy with previously published work that showed NSP2 actually impairs (and not increase) microRNA-mediated silencing. The paper would be of interest to RNA biologists and for molecular virologists that study SARS-CoV- 2.
Response: We thank the reviewer for the positive assessment of our manuscript and acknowledging the importance of our findings.

Reviewer #2
(Evidence, reproducibility and clarity (Required)):
In this study, Naeli and collaborators propose a role for the SARS-CoV2 protein NSP2 in the miRNA-mediated translational repression. Their data show that NSP2 co-immunoprecipitate with the Argonaute AGO2 and the previously reported translation modulator GIGYF2. Using different reporters sensitive to miRNA repression, they show that overexpressing NSP2 enhances miRNA-mediated translational repression in different cell lines as well as one endogenous miRNA target CDK1 and thus without affecting the stability of targeted mRNAs. Interestingly, their data show that the effect of NSP2 depends on the number of miRNA binding sites suggesting that highly repressed mRNAs, due to the presence of several miRNA binding sites, are not affected by NSP2. Finally, using an AGO2 tethering reporter assay that does not rely on miRNA binding but needs two NSP2 interactors GIGYF2 and 4EHP, they demonstrate that overexpressing NSP2 still stimulates mRNA repression, suggesting that NSP2 can have a broader impact on miRISC-dependent silencing.

Overall, this is an interesting follow-up study from their previous paper (Xu et al., 2022) that have the potential to add another dimension to the modulation of mRNA translation by the SARS-CoV2 protein NSP2. But unfortunately, in its current form, the work falls short of supporting their claims appropriately and providing sufficient and relevant insights about the role of NSP2 in regulating the miRNA-mediate gene regulation. To overcome this, the authors should perform and add the following experiments to strengthen their study.

© 2023. Published by The Company of Biologists under the terms of the Creative Commons Attribution License (https://creativecommons.org/licenses/by/4.0/).

Response: We thank the reviewer for the positive assessment of our manuscript, acknowledging the importance of our findings, and insightful suggestions to improve the manuscript.

1- *Interaction of NSP2 with the miRISC: With the data presented here, we can only conclude that NPS2 forms a complex with AGO2. Is their interaction direct or indirect? Is miRNA- and TNRC6-bound AGO2 (and thus miRISC) associated with NSP2? To address those important questions, the authors should perform NSP2 immunoprecipitations in GIGYF2 (and 4EHP) KO cell lines and monitor the presence of AGO2 and miRNAs in the immunoprecipitated complex. Those experiments will define the type of interaction NSP2 has with the miRISC (GIGYF2 dependent or not).*

Response: we thank the reviewer for this constructive comment. To address this, we plan to perform immunoprecipitation/mass-spectrometry (IP/MS) with FLAG-tagged NSP2 and FLAG-GFP control in parental and GIGYF2-KO HEK293 cells to analyse the GIGYF2-dependent interactome of NSP2 with an unbiased approach. If IP/MS fails due to unexpected reasons, we will use western blot to probe for the miRISC components (e.g. AGO2, TNRC6) in parental and GIGYF2-KO cells. We will also use targeted qRT-PCR to quantify candidate miRNAs (Let7, miR-20, and miR-92) in NSP2- pull down eluate in the parental and GIGYF2-KO cells to assess if the NSP2-associated TNRC6 and AGO2 are miRNA-bound.

2- Along this line, is NSP2 action on miRNA-mediated gene regulation dependent or not of GIGYF2? Again, testing this by monitoring the repression of the different reporters upon NSP2 overexpression in the presence or not of GIGYF2 in cells will precise the contribution of NSP2 in miRNA-mediated gene silencing.

Response: We agree with the reviewer that this is an important point and plan to repeat our reporter assays with various miRNA reporters in the GIGYF2-KO cells (in parallel with parental cells) upon NSP2 expression to assess whether the miRNA-mediated gene regulation depends on GIGYF2.

Besides these two sets of critical experiments that will define the relationship between NSP2 mRNA modulation and the microRNA pathway, it would be interesting for this study to readily test the effect of NSP2 on miRNA targets related to immune-regulatory processes and antiviral response. As the authors pointed out in their discussion, several mRNAs are involved in the control of genes found in those pathways. Demonstrating the contribution of NSP2 in the regulation of a few of them will strengthen their study's significance and interest by providing a possible mechanism at play during a SARS-CoV2 infection. Also, it would be interesting to test if the modulation of translation inhibition by NSP2 occurs in cells infected by the SARS-CoV2 virus. For instance, is NSP2 and miRISC interaction enhances during the viral infection? As the authors propose, NSP2 could also have improved antiviral microRNA repression, so it would be interesting to characterize this interplay in a relevant biological context.

Response: We agree with the reviewer that assessing the impact of NSP2 in the context of viral infection is important, and in fact this was the main subject of our recent report, wherein we showed that GIGYF2 and/or 4EHP-depletion hampers SARS-CoV-2 infection due to enhanced IFNß expression and NSP2 increases viral infection by translational repression of the *Ifn1b* mRNA and thereby dampening IFNß production (Zhang et al, PNAS 2022)[10]. However, in the scope of this manuscript, we aimed at defining the role of NSP2, independent of other viral factors, which could have substantial impacts on expression of the various innate immune response genes on their own and therefore could make the interpretation of the impacts of NSP2 very difficult. For instance, NSP1 is known to cause transcriptional shutdown of the *Ifnb1* mRNA as well as inducing general translational repression[11, 12]. Hence, we believe testing the effect of NSP2 in the context of viral infection will not benefit the impact of this manuscript.

Minor comment:

From the data presented in Figure S1A, it is impossible to conclude "that miR-20a represses the expression of the target mRNA in a GIGFY2-dependent manner" as its KO also affect the level/stability of 4EHP. Therefore, we cannot distinguish the contribution of GIGFY2 and 4EHP in

© 2023. Published by The Company of Biologists under the terms of the Creative Commons Attribution License (https://creativecommons.org/licenses/by/4.0/).

this context as both protein levels decrease. The authors should tone down this statement on page 3.

Response: We thank the reviewer for this insightful comment. This is an important point, and we agree with the reviewer that removing GIGYF2 affects 4EHP expression. However, our previous study demonstrated that rescuing expression of 4EHP, which is destabilized in GIGYF2-depleted cells, failed to restore repression of IFN-β production in GIGYF2-KO HEK293 cells (Fig. 3C and D in Zhang et al, PNAS 2022)[10]. This supports our conclusion that the NSP2-induced enhanced repression is GIGYF2-dependent.

Notably, we had also shown that a mutant form of GIGYF2 that does not interact with 4EHP still inhibits translation of the target mRNA, albeit much less effectively (Fig. 3E and F in Zhang et al, PNAS 2022)[10]. This suggests that besides 4EHP, GIGYF2 may have other, yet unknown, partner(s) that repress mRNA translation. It also further supports our conclusion that NSP2-induced enhanced repression is GIGYF2-dependent but only partially 4EHP-dependent. We are currently investigating the nature of the other likely partner(s) of GIGYF2, the results of which will be published in a future manuscript.

Reviewer #2 (Significance (Required)):
With the addition of the proposed experiments, the revised study will better define the direct contribution of NSP2 with the miRISC. This work has the potential to provide another aspect of the mRNA translation modulation by the SARS-CoV2 protein NSP2 with the interesting angle of miRNA- mediated gene silencing.
As mentioned by the authors, another recent paper reports the potential impact of NSP2 on post-transcriptional silencing (Zou et al., iScience 2022). However, in contrast to the current study, this previous work did not directly test the interaction of NSP2 with the miRISC. Furthermore, it only used a single miRNA reporter (let-7) to support NSP2 contribution in miRNA-mediated gene silencing, which does not demonstrate the broader impact of NSP2 on this gene regulatory mechanism as tested in this current study. Upon revision, this study will provide more definitive proof of the involvement of NSP2 in miRNA-mediated gene regulation and thus will be of interest to experts in the miRNA field and scientists interested in understanding viruses/hosts interaction.
Although not essential, if the authors want to add data that addresses the function of NSP2 on this regulatory pathway in the context of viral infection (which seems feasible for this group), that will definitely increase the broader significance of their work.
I am an expert in molecular biology and molecular genetics, miRNA-mediated gene regulation, and small non-coding RNA biology.

Response: We thank the reviewer for the positive assessment of our manuscript and for constructive suggestions. Regarding the comments on *addressing the function of NSP2 on regulatory pathway in the context of viral infection",* please see our response to an earlier and similar comment by this reviewer.

Reviewer #3
(Evidence, reproducibility and clarity (Required)):
The manuscript by Naeli et al., presents study on effects of SARS-CoV-2 NSP2 protein on miRNA mediated translational repression. Authors show in multiple ways using reporter mRNAs with various miRNA sites that NSP2 protein stimulates miRNA mediated repression. The increase in miRNA repression is likely due to the interaction of NSP2 with either GIGYF2 or Argonaute protein directly thus making more stable repressive complex on mRNA. The manuscript is written clearly and methods provide enough details for reproducibility. Only major comment would be that authors could have tested multiple endogenous targets of miRNAs for extent of miRNA mediated expression in the presence of NSP2. This could be achieved using either western blots for known targets (looking at protein levels) or using targeted qRT-PCR or distribution of mRNAs in polysome fractions (mRNA translational repression. An alternative would be Ribo-Seq experiment.

Response: We thank the reviewer for the positive assessment of our manuscript and acknowledging the importance of our findings. This comment is in line with 1st comment of reviewer # 1. To address this, in the planned revision of this manuscript, we will analyse the impact of NSP2 on translation of several known native microRNA target transcripts, such as *Hmga2* and *Ccnd1,* which are known

© 2023. Published by The Company of Biologists under the terms of the Creative Commons Attribution License (https://creativecommons.org/licenses/by/4.0/).

microRNA target mRNAs and our reporter assay (Fig. 3C) and western blot (Fig. 1F) analyses indicates their sensitivity to NSP2-induced repression. We will assess the impact of NSP2 on translation of these native transcripts by analysing their distribution in sucrose gradients.

Minor comment:
Line in introduction arguing: "The GIGYF2/4EHP complex is recruited by a variety of factors including miRNAs" should state miRISC instead of miRNAs.

Response: We thank the reviewer for highlighting this and have now revised the text to correct this mistake.

References:

[1] Jafarnejad SM, Chapat C, Matta-Camacho E, Gelbart IA, Hesketh GG, Arguello M, Garzia A, Kim SH, Attig J, Shapiro M, Morita M, Khoutorsky A, Alain T, Christos GG, Stern-Ginossar N, Tuschl T, Gingras AC, Duchaine TF, Sonenberg N: Translational control of ERK signaling through miRNA/4EHP-directed silencing. Elife 2018, 7.
[2] Zhang X, Chapat C, Wang P, Choi JH, Li Q, Luo J, Wiebe S, Kim SH, Robichaud N, Karam IF, Dai D, Hackett AP, Lin RT, Alain T, Yang L, Jafarnejad SM, Sonenberg N: microRNA-induced translational control of antiviral immunity by the cap-binding protein 4EHP. Mol Cell 2021, 81:1187-+.
[3] Chapat C, Jafarnejad SM, Matta-Camacho E, Hesketh GG, Gelbart IA, Attig J, Gkogkas CG, Alain T, Stern-Ginossar N, Fabian MR, Gingras AC, Duchaine TF, Sonenberg N: Cap-binding protein 4EHP effects translation silencing by microRNAs. P Natl Acad Sci USA 2017, 114:5425-30.
[4] Chen C, Zhu C, Huang J, Zhao X, Deng R, Zhang H, Dou J, Chen Q, Xu M, Yuan H, Wang Y, Yu J: SUMOylation of TARBP2 regulates miRNA/siRNA efficiency. Nat Commun 2015, 6:8899.
[5] Amaya Ramirez CC, Hubbe P, Mandel N, Bethune J: 4EHP-independent repression of endogenous mRNAs by the RNA-binding protein GIGYF2. Nucleic Acids Res 2018, 46:5792-808.
[6] Zekri L, Kuzuoglu-Ozturk D, Izaurralde E: GW182 proteins cause PABP dissociation from silenced miRNA targets in the absence of deadenylation. EMBO J 2013, 32:1052-65.
[7] Eulalio A, Huntzinger E, Izaurralde E: GW182 interaction with Argonaute is essential for miRNA- mediated translational repression and mRNA decay. Nat Struct Mol Biol 2008, 15:346-53.
[8] Lackinger M, Sungur AO, Daswani R, Soutschek M, Bicker S, Stemmler L, Wust T, Fiore R, Dieterich C, Schwarting RKW, Wohr M, Schratt G: A placental mammal-specific microRNA cluster acts as a natural brake for sociability in mice. Embo Rep 2019, 20.
[9] Rasch F, Weber R, Izaurralde E, Igreja C: 4E-T-bound mRNAs are stored in a silenced and deadenylated form. Gene Dev 2020, 34:847-60.
[10] Xu Z, Choi JH, Dai DL, Luo J, Ladak RJ, Li Q, Wang YM, Zhang C, Wiebe S, Liu ACH, Ran XZ, Yang JQ, Naeli P, Garzia A, Zhou LL, Mahmood N, Deng QY, Elaish M, Lin RT, Mahal LK, Hobman TC, Pelletier J, Alain T, Vidal SM, Duchaine T, Mazhab-Jafari MT, Mao XJ, Jafarnejad SM, Sonenberg N: SARS-CoV-2 impairs interferon production via NSP2-induced repression of mRNA translation. P Natl Acad Sci USA 2022, 119.
[11] Lokugamage KG, Narayanan K, Huang C, Makino S: Severe acute respiratory syndrome coronavirus protein nsp1 is a novel eukaryotic translation inhibitor that represses multiple steps of translation initiation. J Virol 2012, 86:13598-608.
[12] Thoms M, Buschauer R, Ameismeier M, Koepke L, Denk T, Hirschenberger M, Kratzat H, Hayn M, Mackens-Kiani T, Cheng J, Straub JH, Sturzel CM, Frohlich T, Berninghausen O, Becker T, Kirchhoff F, Sparrer KMJ, Beckmann R: Structural basis for translational shutdown and immune evasion by the Nsp1 protein of SARS-CoV-2. Science 2020, 369:1249-55.

Original submission

First decision letter

MS ID#: JOCES/2023/261286

MS TITLE: SARS-CoV-2 protein NSP2 enhances microRNA-mediated translational repression

© 2023. Published by The Company of Biologists under the terms of the Creative Commons Attribution License (https://creativecommons.org/licenses/by/4.0/).

AUTHORS: Parisa Naeli, Xu Zhang, Patric Leo Harris Snell, Susanta Chatterjee, Muhammad Kamran, Reese Jalal Ladak, Nick Orr, Thomas F Duchaine, Nahum Sonenberg, and Seyed Mehdi Jafarnejad
ARTICLE TYPE: Research Article

Thank you for submitting your work to JCS. Because all three expert reviewers thought that your work was potentially quite interesting and significant, I encourage you to re-submit a revised version of the manuscript including the additional experiments that you identified in your revision plan.

Please ensure that you clearly highlight all changes made in the revised manuscript. Please avoid using 'Tracked changes' in Word files as these are lost in PDF conversion.

I should be grateful if you would also provide a point-by-point response detailing how you have dealt with the points raised by the reviewers in the 'Response to Reviewers' box. Please attend to all of the reviewers' comments. If you do not agree with any of their criticisms or suggestions please explain clearly why this is so.

First revision

Response to reviewers

We thank the reviewers for their positive assessments and providing constructive comments to greatly improve our manuscript. Following the reviewer suggestions and as outlined in our itemized response, we performed new experiments, revised the text, and modified/added new figures to further support our conclusions. We believe our revised manuscript is now considerably strengthened. Please find below our point-by-point response to the reviewers' comments, in blue.

Reviewer #1
(Evidence, reproducibility and clarity (Required)):
In this manuscript the authors examine whether SARS-CoV-2 protein, NSP2 mediate translation repression of cellular transcripts by increasing miRNA mediated suppression. The same authors previously demonstrated NSP2 interacts with GIGYF2 and this interaction suppresses the translation of the Ifnb mRNA. Here they extend this finding and using a series of reporters they illustrate that at least part of NSP2 translation suppression is mediated by increasing GIGYF2 mediated miRNA translation suppression.
Overall the manuscript is clearly written and the experiments well executed.

Major comments:
Optional- The authors show that the ability of NSP2 to enhance miRNA-mediated repression is dependent on the extent of the initial repression and suggest this dependency on the initial repression levels may explain the discrepancy with published work showing NSP2 actually impairs (and not increase) microRNA-mediated silencing. Therefore, an important addition could be to examine what happen to translation in cells that express NSP2 and whether generally translation repression is significant in transcripts (native) that are enriched in miRNA binding site. This experiment will help to show NSP2 effect on native transcripts and potentially help to understand how much of these changes are likely explained by miRNAs.
Response: We thank the reviewer for this suggestion. To analyse the changes in general mRNA translation by NSP2, we performed polysome profiling assay in HEK293 cells transfected with Flag-NSP2 or Flag-GFP as a control. We observed that NSP2 overexpression does not have an impact on the general mRNA translation in the cells (**Rebuttal Fig. 1A & Fig. S1E in the revised manuscript**). Importantly, this observation is expected. Our previous analyses of the changes in mRNA translation upon depletion of GIGYF2 or 4EHP, the two cellular factors that affect the microRNA-mediated translational repression and form a complex with NSP2, had also shown no apparent change in general mRNA translation[1, 2]. This is likely because these factors affect mRNA translation in a

© 2023. Published by The Company of Biologists under the terms of the Creative Commons Attribution License (https://creativecommons.org/licenses/by/4.0/).

transcript-specific manner, dictated by miRNAs or RBPs that recruit the GIGYF2/4EHP complex to the mRNAs.

To assess the impact of NSP2 on repression of a native transcript mRNA, we assessed its impact on translation of *Ccnd1* mRNA, a known target of miR-20 microRNA[3], as out model mRNA. RT-qPCR analysis of the *Ccnd1* mRNA in cDNA libraries derived from the lightly translated vs highly translated fractions of the sucrose gradient confirmed that overexpression of NSP2 results in a slight, but significant, enrichment of *Ccnd1* mRNA in the light sucrose fractions that are typically enriched for translationally repressed mRNAs (**Rebuttal Fig. 1B & Fig. 1G in the revised manuscript**). This is consistent with the known functions of miRNAs that effect subtle repression of their target mRNAs[4] and our observation that NSP2 reduces the expression of CyclinD1 protein by ~19% (**Fig. 1F &** S1D, E) and enhances repression of a reporter mRNA with miR-20 binding sites (**Fig. 1D**).

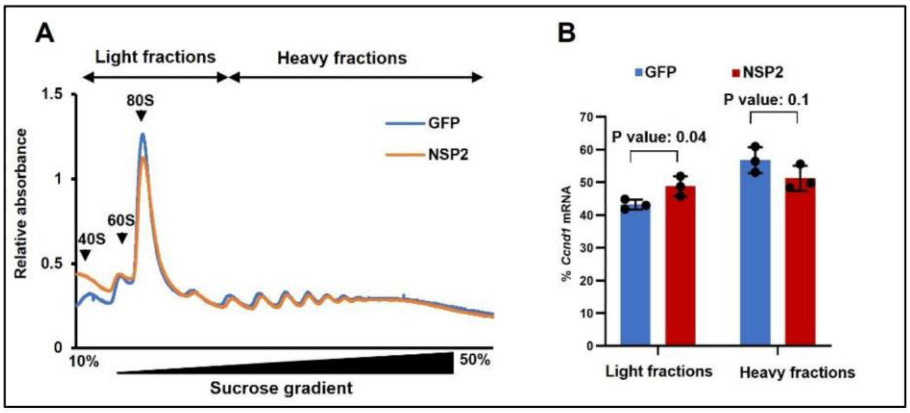

Rebuttal Figure 1. (**A**) Polysome profiling assay in HEK293 cells 24 h after transfection with NSP2 or GFP control. (**B**) RT-qPCR analysis of *Ccnd1* mRNA in sucrose fractions derived from polysome profiling described in A.

Minor comments:

1. Personally I found the term repression fold quite confusing and unintuitive. Why not to present the actual measurements so that the control (blue bars) have high value and the red bars (representing repression) have lower values?

Response: "Repression fold" has been used by us[3] and others[4] in the literature relevant to regulation of mRNA translation due to its effectiveness in visual depiction of relatively smaller changes in repression of mRNA translation, compared with the alternative "expression ratio". For instance, we have compared the two methods of visualisation of the same data (increased repression of Let-7 reporter in HEK293 cells by NSP2; **Rebuttal Fig. 2**), based on which we believe the "Repression fold" method is better in visual depiction of even small changes in repression of the reporter mRNA.

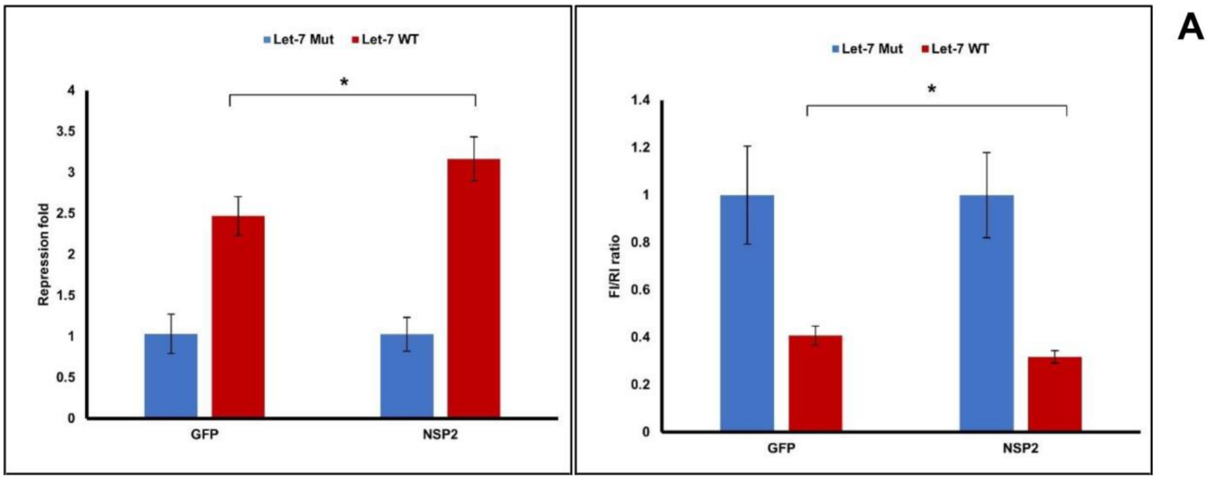

© 2023. Published by The Company of Biologists under the terms of the Creative Commons Attribution License (https://creativecommons.org/licenses/by/4.0/).

Rebuttal Figure 2. NSP2-induced enhanced repression mediated by let-7 depicted using repression fold (**A**) and expression ratio (**B**) methods.

2. It seems inadequate to present on the same graph two values that are normalized to 1. For example, in figure 1D it will be important to show the mir20-Mut real values (at least mir20-Mut in NSP2 cells should not be normalized to 1). This will allow to show that the differences are indeed mediated by stronger translation repression of miR-20 WT luciferase in the presence of NSP2 and not by unexplained differences in mir20-mut luciferase expression. This is true for almost all the figures in the manuscript. Correspondingly, the statistical test should be two-factor ANOVA test examining if NSP2 expression significantly increase the difference between Luciferase miR20-WT and luciferase miR20-mut.

Response: We have used normalization of mutant reporter for this assay, along with two-tailed Student t-test statistical analysis, as it is the common practice in the field[5-9]. In order to compare the outcome between the current method and the one suggested by the reviewer, we performed parallel analysis of 3 experiments with normalized mutant reporter and paired student t-test (Rebuttal Fig. 3 A, C, & E) vs non-normalized mutant reporter and 2-way ANOVA test (Rebuttal Fig. 3B, D, & F), which revealed very similar outcomes (statistically significant NSP2-induced enhanced translational repression induced by miRNAs), regardless of the type of analysis.

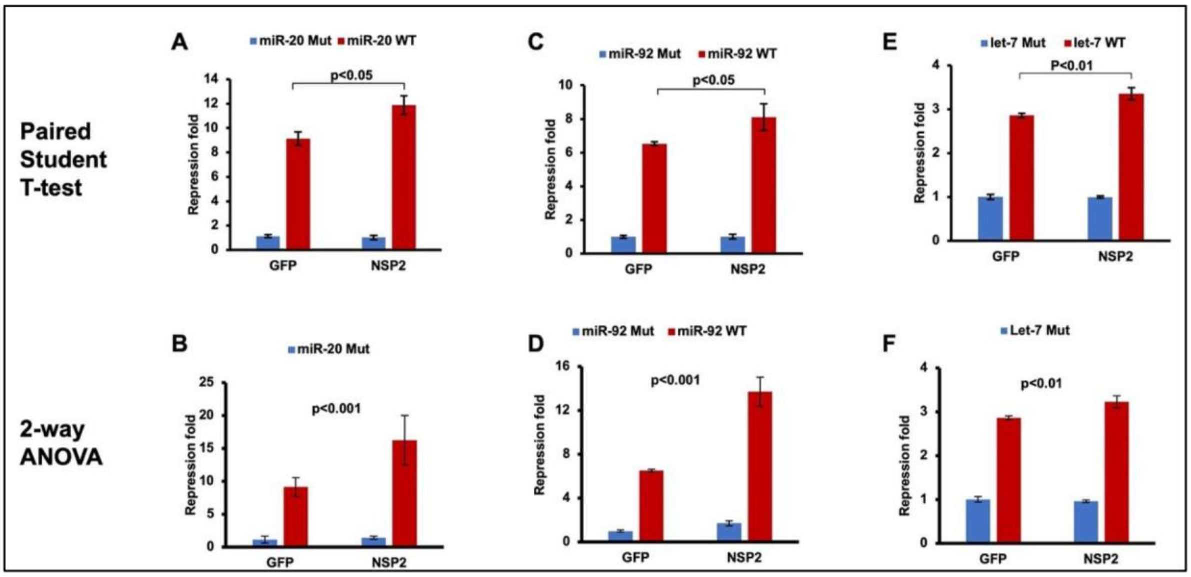

Rebuttal Figure 3. Analysis of the effect of NSP2 on repression induced by miR-20, miR-92, and Let-7 miRNAs in HEK293 cells using paired student t-test (Panels A, C, & E) vs non-normalized mutant reporter and 2-way ANOVA test (Panels B, D, & F).

Reviewer #1 (Significance (Required)):

There is an urgent need for better molecular understanding of how SARS-CoV-2 proteins influence the machineries of the host cell. This study investigates how NSP2 interaction with GIGYF2 mediate translation repression of cellular transcript. The authors also address the discrepancy with previously published work that showed NSP2 actually impairs (and not increase) microRNA-mediated silencing. The paper would be of interest to RNA biologists and for molecular virologists that study SARS-CoV-2.

Response: We thank the reviewer for the positive assessment of our manuscript and acknowledging the importance of our findings.

© 2023. Published by The Company of Biologists under the terms of the Creative Commons Attribution License (https://creativecommons.org/licenses/by/4.0/).

Reviewer #2
(Evidence, reproducibility and clarity (Required)):
In this study, Naeli and collaborators propose a role for the SARS-CoV2 protein NSP2 in the miRNA-mediated translational repression. Their data show that NSP2 co-immunoprecipitate with the Argonaute AGO2 and the previously reported translation modulator GIGYF2. Using different reporters sensitive to miRNA repression, they show that overexpressing NSP2 enhances miRNA-mediated translational repression in different cell lines as well as one endogenous miRNA target CDK1 and thus without affecting the stability of targeted mRNAs. Interestingly, their data show that the effect of NSP2 depends on the number of miRNA binding sites suggesting that highly repressed mRNAs, due to the presence of several miRNA binding sites, are not affected by NSP2. Finally, using an AGO2 tethering reporter assay that does not rely on miRNA binding but needs two NSP2 interactors GIGYF2 and 4EHP, they demonstrate that overexpressing NSP2 still stimulates mRNA repression, suggesting that NSP2 can have a broader impact on miRISC-dependent silencing.
Overall, this is an interesting follow-up study from their previous paper (Xu et al., 2022) that have the potential to add another dimension to the modulation of mRNA translation by the SARS-CoV2 protein NSP2. But unfortunately, in its current form, the work falls short of supporting their claims appropriately and providing sufficient and relevant insights about the role of NSP2 in regulating the miRNA-mediate gene regulation. To overcome this, the authors should perform and add the following experiments to strengthen their study.

Response: We thank the reviewer for the positive assessment of our manuscript, acknowledging the importance of our findings, and insightful suggestions to improve the manuscript.
1-Interaction of NSP2 with the miRISC: With the data presented here, we can only conclude that NPS2 forms a complex with AGO2. Is their interaction direct or indirect? Is miRNA- and TNRC6-bound AGO2 (and thus miRISC) associated with NSP2? To address those important questions, the authors should perform NSP2 immunoprecipitations in GIGYF2 (and 4EHP) KO cell lines and monitor the presence of AGO2 and miRNAs in the immunoprecipitated complex. Those experiments will define the type of interaction NSP2 has with the miRISC (GIGYF2 dependent or not).

Response: We thank the reviewer for this important point. To address this, we performed immunoprecipitation/western blot with Flag-tagged NSP2 and Flag-GFP control in parental and GIGYF2-KO HEK293 cells which showed that depletion of GIGYF2 results in a clear reduction in pull-down of AGO2 with NSP2 (**Rebuttal Figure. 4 and Fig. 1A in the revised manuscript).**
We would like to point out that formation of the complex between GIGYF2 AGO proteins is known to be indirect and enabled by the GYF:PPGL motifs-mediated direct binding of GIGYF2 to the TNRC6 proteins[12]. Therefore, at no point we suggest that NSP2 or GIGYF2 directly bind to AGO2 and our new data (depletion of AGO2 pulldown by NSP2 in GIGYF2-KO cells) further support that model, which is presented in **Figure 5**. We further clarified this in the first paragraph of the result section of the revised manuscript: "This indicates that the interaction between NSP2 and AGO2 is indirect and mediated by GIGYF2, which was previously shown to directly bind to NSP2[13] as well as the TNRC6 subunit of miRISC[12]."

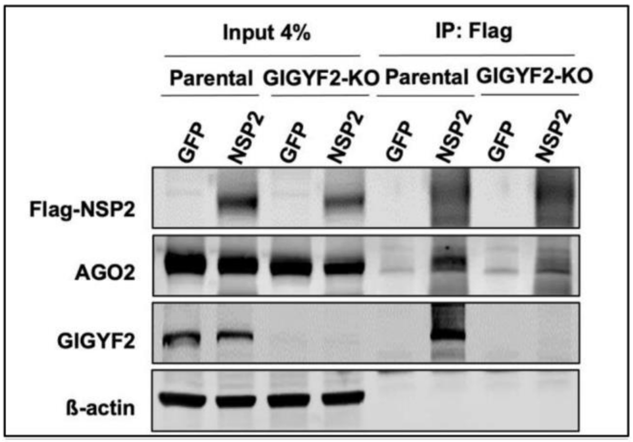

© 2023. Published by The Company of Biologists under the terms of the Creative Commons Attribution License (https://creativecommons.org/licenses/by/4.0/).

Rebuttal Figure 4. Parental and GIGYF2-KO HEK293 cells were co-transfected with Flag-GFP or Flag-NSP2 plasmids. 24 h later, cell lysates were immunoprecipitated using anti-Flag antibody and western blot was performed with the specified antibodies.

2- Along this line, is NSP2 action on miRNA-mediated gene regulation dependent or not of GIGYF2? Again, testing this by monitoring the repression of the different reporters upon NSP2 overexpression in the presence or not of GIGYF2 in cells will precise the contribution of NSP2 in miRNA-mediated gene silencing.

Response: We agree with the reviewer that this is an important point. To address this, we performed a tether function assay with AGO2 in the parental, GIGYF2-KO, and 4EHP-KO HEK293 cells. Our results demonstrate that while, as expected, in parental cells NSP2 expression enhances the AGO2-mediated repression of the deadenylation-resistant *RL-5BoxB-HhR* reporter, GIGYF2 or 4EHP knockout abrogate this effect of NSP2 on AGO2-mediated repression (**Rebuttal Figure. 5 and Fig. 4E in the revised manuscript**). This clearly indicates that NSP2 action on miRNA-mediated gene regulation is GIGYF2-dependent.

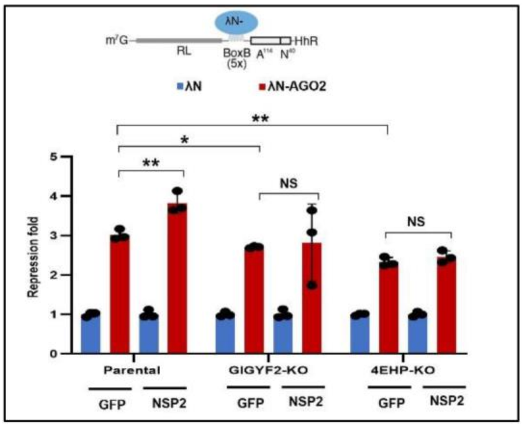

Rebuttal Figure 5. Parental, GIGYF2-KO, and 4EHP-KO HEK293 cells were co-transfected with the RL-5BoxB-HhR reporter and λN-AGO2 or λN-empty plasmid, along with FL plasmid (control) and either Flag- NSP2 or Flag-GFP. RL and FL luciferase activity was measured 24 h after transfection and RL values were normalized against FL levels. Repression fold was calculated for the λN-AGO2 relative to λN-empty level for each condition. Data are presented as mean ± SD (n=3). *$p<0.05$; **$p<0.01$; ***$p<0.001$.

Besides these two sets of critical experiments that will define the relationship between NSP2 mRNA modulation and the microRNA pathway, it would be interesting for this study to readily test the effect of NSP2 on miRNA targets related to immune-regulatory processes and antiviral response. As the authors pointed out in their discussion, several mRNAs are involved in the control of genes found in those pathways. Demonstrating the contribution of NSP2 in the regulation of a few of them will strengthen their study's significance and interest by providing a possible mechanism at play during a SARS-CoV2 infection. Also, it would be interesting to test if the modulation of translation inhibition by NSP2 occurs in cells infected by the SARS-CoV2 virus. For instance, is NSP2 and miRISC interaction enhances during the viral infection? As the authors propose, NSP2 could also have improved antiviral microRNA repression, so it would be interesting to characterize this interplay in a relevant biological context.

Response: We agree with the reviewer that assessing the impact of NSP2 in the context of viral infection is important, and in fact this was the main subject of our recent report[10], wherein we showed that GIGYF2 and/or 4EHP-depletion hampers SARS-CoV-2 infection due to enhanced IFN-ß expression and that NSP2 increases viral infection by translational repression of the *Ifn1b* mRNA and thereby dampening IFNß production. However, in the scope of this manuscript, we aimed at defining the role of NSP2, independent of other viral factors, which could have substantial impacts on expression of the various innate immune response genes on their own and therefore could make the interpretation of the stand-alone impacts of NSP2 impossible. For instance, NSP1 is known to cause transcriptional shutdown of the *Ifnb1* mRNA as well as inducing general translational

© 2023. Published by The Company of Biologists under the terms of the Creative Commons Attribution License (https://creativecommons.org/licenses/by/4.0/).

repression[11, 12]. Hence, we believe testing the effect of NSP2 in the context of viral infection will not benefit the impact of this manuscript.

Minor comment:

From the data presented in Figure S1A, it is impossible to conclude "that miR-20a represses the expression of the target mRNA in a GIGFY2-dependent manner" as its KO also affect the level/stability of 4EHP. Therefore, we cannot distinguish the contribution of GIGFY2 and 4EHP in this context as both protein levels decrease. The authors should tone down this statement on page 3.

Response: We thank the reviewer for this insightful comment. This is an important point, and we agree with the reviewer that removing GIGYF2 affects 4EHP expression. However, our previous study demonstrated that rescuing expression of 4EHP, which is destabilized in GIGYF2-depleted cells, failed to restore repression of IFN-ß production in GIGYF2-KO HEK293 cells (Fig. 3C and D in Zhang et al, PNAS 2022)[10]. This supports our conclusion that the NSP2-induced enhanced repression is GIGYF2-dependent.
Notably, we had also shown that a mutant form of GIGYF2 that does not interact with 4EHP still inhibits translation of the target mRNA, albeit much less effectively (Fig. 3E and F in Zhang et al, PNAS 2022)[10]. This suggests that besides 4EHP, GIGYF2 may have other, yet unknown, partner(s) that repress mRNA translation. It also further supports our conclusion that NSP2-induced enhanced repression is GIGYF2-dependent but only partially 4EHP-dependent. We are currently investigating the nature of the other likely partner(s) of GIGYF2, the results of which will be published in a future manuscript.

Reviewer #2 (Significance (Required)):
With the addition of the proposed experiments, the revised study will better define the direct contribution of NSP2 with the miRISC. This work has the potential to provide another aspect of the mRNA translation modulation by the SARS-CoV2 protein NSP2 with the interesting angle of miRNA-mediated gene silencing.
As mentioned by the authors, another recent paper reports the potential impact of NSP2 on post-transcriptional silencing (Zou et al., iScience 2022). However, in contrast to the current study, this previous work did not directly test the interaction of NSP2 with the miRISC. Furthermore, it only used a single miRNA reporter (let-7) to support NSP2 contribution in miRNA-mediated gene silencing, which does not demonstrate the broader impact of NSP2 on this gene regulatory mechanism as tested in this current study. Upon revision, this study will provide more definitive proof of the involvement of NSP2 in miRNA-mediated gene regulation and thus will be of interest to experts in the miRNA field and scientists interested in understanding viruses/hosts interaction.
Although not essential, if the authors want to add data that addresses the function of NSP2 on this regulatory pathway in the context of viral infection (which seems feasible for this group), that will definitely increase the broader significance of their work.
I am an expert in molecular biology and molecular genetics, miRNA-mediated gene regulation, and small non-coding RNA biology.

Response: We thank the reviewer for the positive assessment of our manuscript and for constructive suggestions. Regarding the comments on *addressing the function of NSP2 on regulatory pathway in the context of viral infection",* please see our response to an earlier and similar comment by this reviewer.

Reviewer #3
(Evidence, reproducibility and clarity (Required)):
The manuscript by Naeli et al., presents study on effects of SARS-CoV-2 NSP2 protein on miRNA mediated translational repression. Authors show in multiple ways using reporter mRNAs with various miRNA sites that NSP2 protein stimulates miRNA mediated repression. The increase in miRNA repression is likely due to the interaction of NSP2 with either GIGYF2 or Argonaute protein directly thus making more stable repressive complex on mRNA. The manuscript is written clearly and methods provide enough details for reproducibility. Only major comment would be that authors could have tested multiple endogenous targets of miRNAs for extent of miRNA mediated expression in the presence of NSP2. This could be achieved using either western blots for known

© 2023. Published by The Company of Biologists under the terms of the Creative Commons Attribution License (https://creativecommons.org/licenses/by/4.0/).

targets (looking at protein levels) or using targeted qRT-PCR or distribution of mRNAs in polysome fractions (mRNA translational repression. An alternative would be Ribo-Seq experiment.

Response: We thank the reviewer for the positive assessment of our manuscript and acknowledging the importance of our findings. This comment similar with the 1st comment of reviewer # 1, which is addressed above (**Rebuttal Fig. 1B & Fig. 1G in the revised manuscript**).

Minor comment:
Line in introduction arguing: "The GIGYF2/4EHP complex is recruited by a variety of factors including miRNAs" should state miRISC instead of miRNAs.

Response: We thank the reviewer for highlighting this and have now revised the text to correct this mistake.

Reviewer #3 (Significance (Required)): *General assessment: The study presents sold evidence that NSP2 protein interacts with miRISC complex and increases miRNA-mediated translational repression of reporter mRNAs. Multiple target sites for miR20, let7 and miR92 are tested as well as two different human cell lines (Hek293 and U87) which gives strength to study and reproducibility of the results. Focus on the endogenous miRNA targets in the presence or absence of the NSP2 protein would make study even stronger. The advance of the study is more rigorous analyses of NSP2 protein effects on miRNA-mediated gene expression regulation with some novel mechanistic insights. The study will be of interest for specialized and basic research audience with potential impact on translational research. My field of expertise covers mechanisms of gene expression regulation by miRNAs and RBPs as well as impact of mRNAs, nascent peptides and ribosomes on protein synthesis.*

Response: We thank the reviewer for the positive assessment of our manuscript and for constructive suggestions.

References:

[1] Jafarnejad SM, Chapat C, Matta-Camacho E, Gelbart IA, Hesketh GG, Arguello M, Garzia A, Kim SH, Attig J, Shapiro M, Morita M, Khoutorsky A, Alain T, Christos GG, Stern-Ginossar N, Tuschl T, Gingras AC, Duchaine TF, Sonenberg N: Translational control of ERK signaling through miRNA/4EHP-directed silencing. Elife 2018, 7.
[2] Zhang X, Chapat C, Wang P, Choi JH, Li Q, Luo J, Wiebe S, Kim SH, Robichaud N, Karam IF, Dai D, Hackett AP, Lin RT, Alain T, Yang L, Jafarnejad SM, Sonenberg N: microRNA-induced translational control of antiviral immunity by the cap-binding protein 4EHP. Mol Cell 2021, 81:1187-+.
[3] Chapat C, Jafarnejad SM, Matta-Camacho E, Hesketh GG, Gelbart IA, Attig J, Gkogkas CG, Alain T, Stern-Ginossar N, Fabian MR, Gingras AC, Duchaine TF, Sonenberg N: Cap-binding protein 4EHP effects translation silencing by microRNAs. P Natl Acad Sci USA 2017, 114:5425-30.
[4] Chen C, Zhu C, Huang J, Zhao X, Deng R, Zhang H, Dou J, Chen Q, Xu M, Yuan H, Wang Y, Yu J: SUMOylation of TARBP2 regulates miRNA/siRNA efficiency. Nat Commun 2015, 6:8899.
[5] Amaya Ramirez CC, Hubbe P, Mandel N, Bethune J: 4EHP-independent repression of endogenous mRNAs by the RNA-binding protein GIGYF2. Nucleic Acids Res 2018, 46:5792-808.
[6] Zekri L, Kuzuoglu-Ozturk D, Izaurralde E: GW182 proteins cause PABP dissociation from silenced miRNA targets in the absence of deadenylation. EMBO J 2013, 32:1052-65.
[7] Eulalio A, Huntzinger E, Izaurralde E: GW182 interaction with Argonaute is essential for miRNA-mediated translational repression and mRNA decay. Nat Struct Mol Biol 2008, 15:346-53.
[8] Lackinger M, Sungur AO, Daswani R, Soutschek M, Bicker S, Stemmler L, Wust T, Fiore R, Dieterich C, Schwarting RKW, Wohr M, Schratt G: A placental mammal-specific microRNA cluster acts as a natural brake for sociability in mice. Embo Rep 2019, 20.
[9] Rasch F, Weber R, Izaurralde E, Igreja C: 4E-T-bound mRNAs are stored in a silenced and deadenylated form. Gene Dev 2020, 34:847-60.
[10] Xu Z, Choi JH, Dai DL, Luo J, Ladak RJ, Li Q, Wang YM, Zhang C, Wiebe S, Liu ACH, Ran XZ, Yang JQ, Naeli P, Garzia A, Zhou LL, Mahmood N, Deng QY, Elaish M, Lin RT, Mahal LK, Hobman TC, Pelletier J, Alain T, Vidal SM, Duchaine T, Mazhab-Jafari MT, Mao XJ, Jafarnejad SM, Sonenberg N: SARS-CoV-2 impairs interferon production via NSP2-induced repression of mRNA translation. P Natl Acad Sci USA 2022, 119.

© 2023. Published by The Company of Biologists under the terms of the Creative Commons Attribution License (https://creativecommons.org/licenses/by/4.0/).

[11] Lokugamage KG, Narayanan K, Huang C, Makino S: Severe acute respiratory syndrome coronavirus protein nsp1 is a novel eukaryotic translation inhibitor that represses multiple steps of translation initiation. J Virol 2012, 86:13598-608.
[12] Thoms M, Buschauer R, Ameismeier M, Koepke L, Denk T, Hirschenberger M, Kratzat H, Hayn M, Mackens-Kiani T, Cheng J, Straub JH, Sturzel CM, Frohlich T, Berninghausen O, Becker T, Kirchhoff F, Sparrer KMJ, Beckmann R: Structural basis for translational shutdown and immune evasion by the Nsp1 protein of SARS-CoV-2. Science 2020, 369:1249-55.

Second decision letter

MS ID#: JOCES/2023/261286
MS TITLE: SARS-CoV-2 protein NSP2 enhances microRNA-mediated translational repression
AUTHORS: Parisa Naeli, Xu Zhang, Patric Leo Harris Snell, Susanta Chatterjee, Muhammad Kamran, Reese Jalal Ladak, Nick Orr, Thomas F Duchaine, Nahum Sonenberg, and Seyed Mehdi Jafarnejad
ARTICLE TYPE: Research Article

Thank you for sending your manuscript to Journal of Cell Science through Review Commons.

I am happy to tell you that your manuscript has been accepted for publication in Journal of Cell Science, pending standard ethics checks. Where referee reports on this version are available, they are appended below.

© 2023. Published by The Company of Biologists under the terms of the Creative Commons Attribution License (https://creativecommons.org/licenses/by/4.0/).
